# Metal Ion Isotope Ratio Using ESI-Orbitrap HRMS: Proof of Concept and Initial Performance Evaluation for Lead Isotopic Ratios

**DOI:** 10.1021/acs.analchem.5c01033

**Published:** 2025-06-17

**Authors:** Gianluca Roncoroni, Davide Spanu, Gilberto Binda, Damiano Monticelli

**Affiliations:** 189767 Università degli Studi dell’Insubria, Dipartimento di Scienza e Alta Tecnologia, via Valleggio 11, Como 22100, Italy

## Abstract

This study introduces a novel approach using an electrospray source coupled to an Orbitrap MS instrument to determine metal isotope ratios. The procedure involves forming a complex between the ion of interest and an appropriate ligand, generating gas-phase ions via electrospray ionization, selecting the complex mass by quadrupole filtering, and performing collisional fragmentation to yield free metal ions. The isotopic pattern of the free ion is then analyzed by high-resolution MS. The approach ensures high selectivity and interference-free spectra. A proof-of-concept study was conducted to determine Pb isotope ratios, focusing on identifying the factors that influence the accuracy and precision of the procedure. At this early stage, optimal accuracy was achieved even in the presence of matrix components by applying mass bias correction methods originally developed for other isotope ratio techniques; precision is comparable to that achieved by single-collector ICP-MS instrumentation. This approach may complement conventional methods that suffer from limited mass resolution and usually require extensive sample preparation.

## Graphical abstract

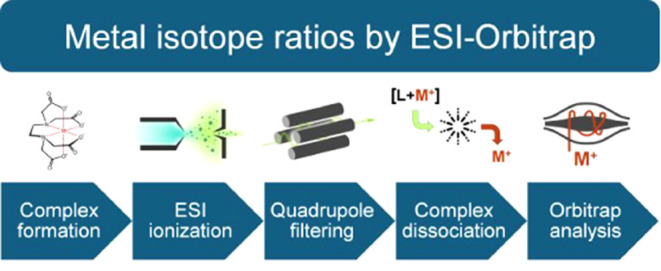

## Introduction

The study of isotopes represents a fascinating world, providing access to immensely useful information often unavailable by other means,^1^ with applications ranging from geochemistry and cosmochemistry,^2^ pollutant studies,^3^ ecology^4^ and food tracing,^5^ to forensic sciences,^6^ etc.

Reliable information can only be achieved when isotope abundances are determined with extreme precision and accuracy. Mass spectrometry, preceded by thermal ionization mass spectrometry (TIMS) or inductively coupled plasma ionization-mass spectrometry (ICP-MS), is the technique of choice for metal isotope studies in solution, while laser ablation (LA) can be coupled to ICP-MS for direct isotopic analysis of solids. TIMS is considered the gold standard in terms of precision and accuracy, but it shows limitations such as low ionization efficiency and low sample throughput and a lack of versatility; it requires intricate and time-consuming sample pretreatment to isolate a specific analyte.^7^ In contrast, ICP-MS benefits from direct liquid sample analysis and higher throughput,^8^ but to obtain high-precision isotope ratios (IRs) with a Multicollector (MC) ICP-MS, the separation of the analyte from the matrix and interfering elements, using one or more chromatographic separation step(s),^9^ is mandatory. On the other hand, matrix separation is not required when using a single-collector ICP-MS, which is more tolerant to matrix differences due to its intrinsically more modest IR precision, or when using LA as a sample introduction system for MC-ICP-MS. Moreover, both TIMS and ICP-MS inherently show low resolution; accordingly, resolving isobaric interferences is often impossible, requiring preanalytical separation and interference correction.^10^ Alternatively, isobaric interferences may be resolved during ICP-MS analysis by selective ion–molecule reactions.^11,12^ Reactive gases like O_2_, CO_2_, N_2_O, NH_3_, or CH_3_F are used to shift the *m*/*z* of the interfering or analyte ion, thus avoiding overlap.

The introduction of the high-field ion trap mass analyzer, commercialized under the name Orbitrap^13,14^ promised to further contribute to IR determination due to its ultrahigh resolution, simultaneous mass spectra acquisition, automation potential, and rapid analysis.^15,16^ Recently, the isotopic composition of stable oxyanions, such as nitrates,^17,18^ sulfates,^17^ and phosphates,^17^ was analyzed using an electrospray ionization (ESI) source, followed by detection through a quadrupole—Orbitrap instrumentation. Nevertheless, ESI is not an efficient source for free metal ions,^19^ and a glow discharge (GD) source was employed (liquid sampling-atmospheric pressure glow discharge (LS-APGD), as introduced in 2011 by Marcus et al.^20^ This microplasma, with a power density of ∼50 W mm^–3^, is mounted as a replacement for the conventional ESI source and has been shown to produce elemental spectra for many ions in acidic aqueous solutions.^21^ The procedure is at the research stage, with no application to samples; it is likely that the need to modify the source remains the main limitation to its widespread adoption.

Here, we propose an analytical strategy to determine the isotopic ratios of metal ions using ESI-Orbitrap instrumentation, employing an autosampler and HPLC pump to transfer the sample to the ESI, though no chromatographic column is used. To overcome the limited ionization efficiency for free metal ions, metal complexes are fed to the ESI source.^22–24^ Then, quadrupole filtering is used to select the complex­(es), followed by collisional dissociation to yield the free isotopes of the metal ion,^25,26^ and finally, the high-resolution mass spectrometer is used to analyze the element(s) isotopes. Elemental mass spectra are obtained as the output, leading to clean and uncomplicated data, avoiding complex deconvolution algorithms to separate the isotopic contributions of the analyte and the ligand.^27^ The high resolution, speed, and multiplexing capability (allowing for the measurement of multiple isotopic ratios in a single scan) provided by Orbitrap indicate reasonable prospects for this procedure, as demonstrated here for the determination of lead isotopic ratios.

## Methods

### Reagents, Solutions, and Standards

Established procedures for solution and standard preparation were adopted (see Supporting Information). LCMS-grade organic solvents and ultrapure water were employed. Method validation and evaluation of mass bias correction methods were conducted on solutions obtained from the standard reference materials 981 and 982 from NIST.

### Instrumentation

All of the analyses were performed using an Orbitrap Exploris 120 (Thermo Scientific) equipped with an OptaMax NG ion source and a Heated-ESI probe (Thermo Scientific). The instrumentation features a quadrupole for MS^1^ selection and a collision cell, named the HCD cell (high-energy collisional dissociation), followed by the Orbitrap mass analyzer. Isotope spectra are acquired after collisional dissociation in MS^2^; details are provided in the Method Outline section, and instrumental parameters are listed in Table S1. The mass spectrometer was coupled to a Vanquish Core UHPLC system and a Vanquish Autosampler model VC-A12-A (Thermo Scientific) equipped with a 1000 μL sample loop. The LC equipment was used only to deliver samples to the mass spectrometer, and no chromatographic column was installed; the line exiting the injection valve was directly fed into the ESI probe. Figure S1 shows a typical chromatogram obtained by using the optimized procedure.

## Results and Discussion

### Method Outline

The present method converts HPLC – ESI – Quadrupole – Orbitrap into an efficient procedure for isotope ratio determination, without introducing any hardware modifications. The rationale and the unique features of each step are described in the following sections (Figure 1).

**Figure 1:**
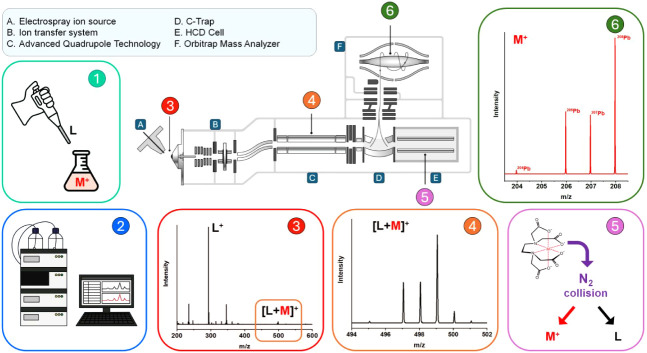
Schematic illustration of the major components of a Thermo Scientific Orbitrap Exploris 120 Mass Spectrometer and their use in the presented procedure. L: ligand; M: metal. Adapted with permission from Orbitrap Exploris Series Orbitrap Exploris 120, Orbitrap Exploris 240, Orbitrap Exploris 480, and Orbitrap Exploris MX Operating Manual, BRE0014471 Revision E October 2021. Copyright 2021 Thermo Fisher Scientific Inc.

#### Step 1: Metal Ion – Ligand Complex Formation

The direct introduction of metal ions in the ESI source resulted in low sensitivity and adduct-rich elemental spectra,^19^ prompting the use of glow discharge (GD) as an efficient source for elements.^20^ Here, we propose the use of metal ion– complex­(es) as they are efficiently transferred into the gas phase by ESI, providing good sensitivity compared to free metal ions. In addition, this strategy offers diverse selectivity mechanisms. The choice of the ligand and the solution pH enables the selective complexation of one or a class of metal ions, whereas ligands of different masses may be employed to shift the *m*/*z* value of the [L + M]^+^ ion to a less interfered zone of the mass spectra, in case interfering species are present.

#### Step 2: Automated Sample Introduction

The measurements are fully automated by using the HPLC system coupled to an autosampler: chromatographic separation is not needed, and the column is bypassed. A common XY autosampler may be better suited, although the latter has never been coupled to an ESI source, to the best of our knowledge.

#### Step 3: Generation of the Gas Phase Ions in the ESI Source

The complexes in solution are ionized and transferred to the gas phase efficiently (Figure 1, box 3) compared to noncomplexed metal ions; preliminary tests showed no Pb signal in MS^1^ in the absence of the complexing agent. The latter efficiency is demonstrated by the intense signals produced in MS^2^ (Zn excluded), typically 300–900k counts/μM (Figure 2). The use of ion complex­(es) and a soft ionization source also avoids the formation of metal ion adducts, such as oxides, nitrates, etc.,^21^ which were not detected in the mass spectrum.

**Figure 2:**
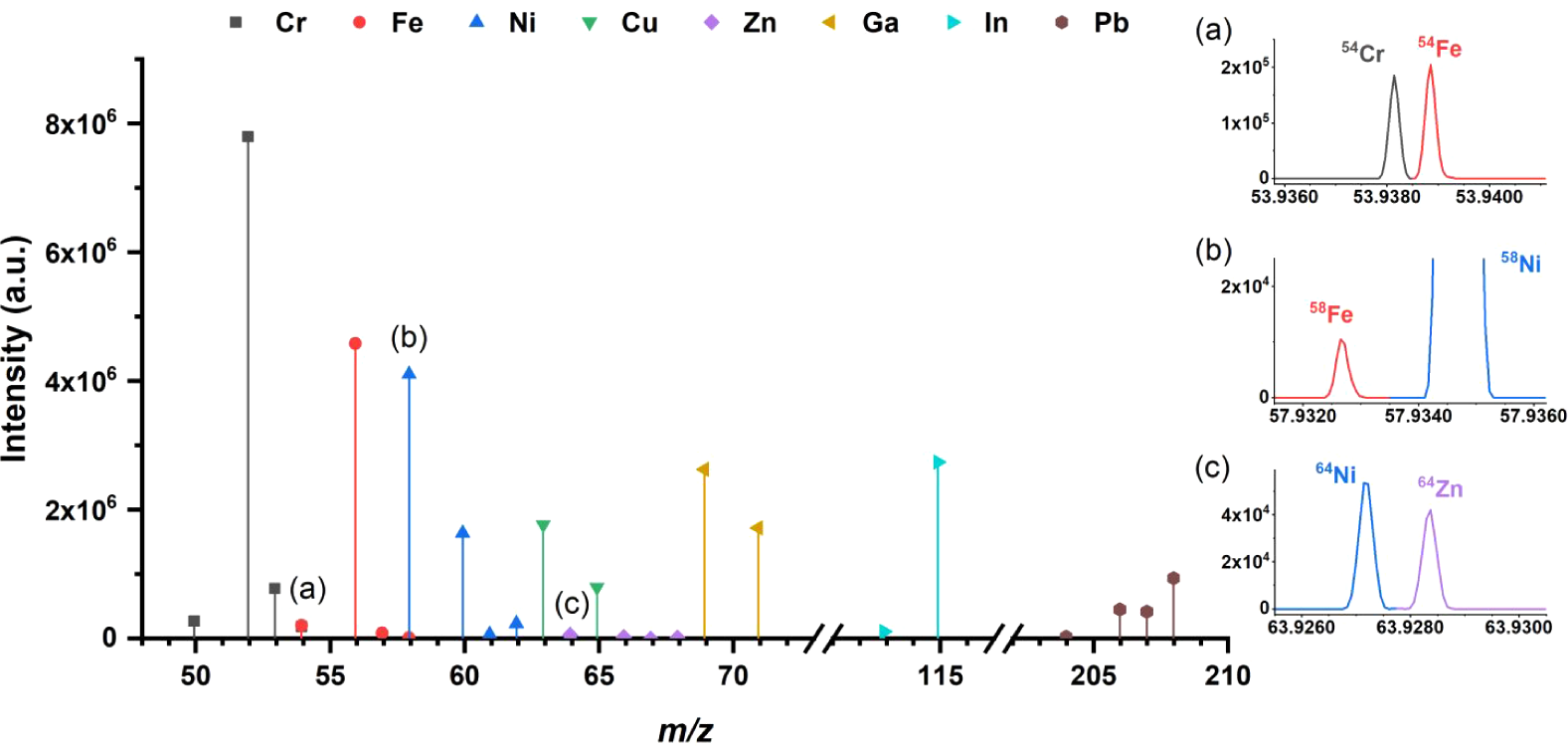
Mass spectra of eight elements, 500 μg/L for each element. Cr, Fe, Ni, Cu, Zn, and Ga were acquired in a single run, and In and Pb were acquired separately. The insets show zoomed-in portions of the mass spectrum demonstrating resolution of the isobaric interferences for the following isotopic pairs: (a) ^54^Cr–^54^Fe; (b) ^58^Fe–^58^Ni; and (c) ^64^Ni–^64^Zn. Resolution was set at 120k@*m*/*z* 200, and calculated from the spectrum as 230k in the *m*/*z* interval 50–71. The masses of the investigated isotopes are recognized with an error lower than 1.76 ppm; see Table S2. Measurement duration: 10 min. Measurement conditions: EDTA: 200 μM. Direct injection flow: 5 μL/min. Solvent: H_2_O:MeOH 1:1. pH: 4.20. Resolution: 120k. Microscan: 10. Quad. Isolation window: 338–368 *m*/*z* (Cr, Fe, Ni, Cu, Zn, and Ga), 397–407 *m*/*z* (In), 490–502 *m*/*z* (Pb). AGC target: 10^6^ charges (Cr, Fe, Ni, Cu, Zn, Ga; In); 10^5^ charges (Pb). Max. injection time: 100 ms. Injection control: AGC (Cr, Fe, Ni, Cu, Zn, Ga; In); time (Pb). HCD: 200%. Scan window: 48–71.5 *m*/*z* (Cr, Fe, Ni, Cu, Zn, and Ga), 112–117 *m*/*z* (In), 203.5–208.5 *m*/*z* (Pb). See Table 1 for parameter definitions.

#### Step 4: Quadrupole Mass Filtering and Ion Trapping

The complex of interest is isolated from the ion mixture produced in the source by the quadrupole and is transmitted to the C-Trap (Figure 1, box 4). The quadrupole isolation window should be precisely wide enough to extract the entire isotopic pattern of the precursor complex (i.e., preserve the isotopic information) but not wider, to avoid the entry of possible interfering ions into the mass analyzer. This step, as is typical in MS^2^ experiments, strongly enhances the signal-to-noise (S/N) ratio by removing most of the nonanalyte ions (matrix components, free ligand, etc.), which may directly interfere with the detection of the analyte (e.g., fragments that fall into the element detection window) or significantly contribute to the ions entering the C-Trap. Reducing the *m*/*z* window width increases the accumulation of the ion of interest at the expense of the interfering species. The C-Trap is filled until it reaches either the target ion accumulation value or the maximum injection time (see Method Development and Key Parameterssection for further details).

#### Step 5: Dissociation of the Metal–Ligand Complex in MS^2^

The ion packet exiting the C-Trap is accelerated within the higher-energy collisional dissociation (HCD) cell. The HCD cell consists of a straight multipole mounted inside a collision gas-filled tube. A voltage offset between the C-Trap and the HCD cell accelerates parent ions into the HCD cell, and a collision with nitrogen causes fragmentation into product ions. Inside the HCD cell, metal ions are freed from the complex, forming +1 gas-phase ions (Figure 1, box 5). This is the key step in directly measuring the isotopic pattern of the free element, avoiding interference from the superimposed isotopic pattern of the ligand. The collision energy should be selected to maximize the yield of free metal ions: though higher acceleration voltages promote fragmentation, it is unlikely that interfering species may produce fragments with *m*/*z* in the element detection window as quadrupole filtering removes most of the nonanalyte ions. In such a case, a compromise between efficient metal ion formation and interfering ion fragment generation should be adopted.

#### Step 6: Measuring Element Isotopic Pattern Simultaneously at High Resolution

The isotopic abundances of the metal ions are measured using the Orbitrap analyzer (Figure 1, box 6). The mass spectrum is simply the isotopic pattern of the element(s) showing *z* = +1 and does not show any relevant interfering peaks (see Figure 2). Advantages of this kind of mass analyzer include the simultaneous detection of all the isotopes of interest, which avoids mass bias due to signal fluctuations in sequential measurements. Additionally, the isotopic pattern of more than one element may be analyzed simultaneously, as shown in Figure 2 for the first-row transition elements. The isotopic patterns, obtained under nonoptimized measurement conditions and not corrected for mass bias, show good agreement with the natural abundances for most of the elements, though some exceptions are evident (Table S2). Zinc IRs strongly deviate from the expected values due to the very low registered signals (see also below). The lowest mass elements, namely Cr and Fe, also showed higher mass bias, possibly because they are close to the 40 *m*/*z* limit of the spectrometer, though this hypothesis remains unverified at present. These data (Figure 2 and Table S2) suggest that the proposed method could be applied to several analytes, provided that adequate optimization is achieved (see the following section). The reason Zn exhibits much lower sensitivity compared to the other elements is not clear. However, it is possible that EDTA is not the optimal ligand for this element due to limitations in the efficiency of gas-phase ion production in the ESI source. Soft ligands may offer a viable alternative for addressing this issue. Furthermore, the detection at ultrahigh resolution, ≈230k in the 50–69 *m*/*z* range, is a unique feature of the present method. It allows for resolving all the isobaric interferences in the first transition row elements, like ^54^Cr– ^54^Fe, ^58^Fe–^58^Ni, and ^64^Ni–^64^Zn (see insets a, b, and c in Figure 2) or ^50^Cr–^50^V and ^50^Ti–^50^Cr (approximately 45k required theoretical resolution, not shown here). In addition, spectra are acquired at a fast rate of approximately 9 microscans per second (see Table 1 for the definition of microscan), improving counting statistics with minimal sample consumption. The proposed strategy accordingly provides interference-free isotope signals that may be directly used for IR calculation.

### Determination of Pb Isotopic Ratios

#### Method Development and Key Parameters

The determination of Pb isotopic ratios is a perfect benchmark for a novel isotope ratio methodology as Pb shows four stable isotopes (^204^Pb, ^206^Pb, ^207^Pb, and ^208^Pb), spanning four mass units with significantly different natural abundances (1.4%, 24.1%, 22.1%, and 52.4%, respectively.^28^ It also exhibits a complex fractionation behavior.^29^ Moreover, the radiogenic nature of the ^206^Pb, ^207^Pb, and ^208^Pb isotopes makes the IR variations particularly informative.^30^ As already reported in Figure 2, the isotopic pattern of Pb in the Pb-EDTA complex is perfectly retained in the MS^1^ spectrum (Figure S2 and Table S3).

The influence of instrumental and chemical parameters on mass bias and precision is initially evaluated, leading to the determination of optimal parameter settings. Table 1 lists the tested instrumental parameters and summarizes their effect on mass bias and precision (see the Supporting Information for further information about the optimization procedure). A typical Pb isotope mass spectrum under optimized conditions is reported in Figure S3. This analysis serves as a foundational investigation; the univariate approach may obscure potential interactions between parameters, warranting further exploration in future studies. Available data suggest that precision is regulated by signal acquisition time, whereas more subtle, still unknown reasons are involved in mass bias changes.

The procedure is finally validated under optimized conditions by evaluating its performance, including an initial assessment of the mass-bias correction methods.

#### Validation

### Accuracy

Isobaric interferences may lead to inaccurate results due to the systematic effect on the signal(s) of one or more analyte isotope(s). In the case of Pb, the isobaric interference of ^204^Hg on ^204^Pb cannot be resolved by the highest resolution provided by our instrumentation (120k); achieving proper separation would require a theoretical resolution of approximately 500k. Nevertheless, the complex between Hg and EDTA is not visible in the MS spectrum under the investigated conditions (200 μM EDTA, pH 4.3, 1:1 H_2_O:MeOH, Hg concentration up to 10 μg/L), prompting the choice of an appropriate ligand as an additional source of selectivity complementary to the instrumental one.

On the other hand, fractionation phenomena during Orbitrap analysis have been demonstrated to affect the accuracy of IR determination, as exemplified by space charge effects^31^, e.g., signal coalescence.^16^ Up to now, standard-sample bracketing (SSB)^18^ and internal standardization^32^ have only been implemented in Orbitrap IR measurements, and matrix-induced fractionation has never been assessed. A simple SSB strategy was demonstrated to efficiently correct the mass bias when the sample and standard matrices match. In this study, the Pb IRs of 500 μg/L Pb NIST SRM 981 or Pb NIST SRM 981 + Pb NIST SRM 982 solutions were corrected between 40 and 370 ppm of their expected values using standard bracketing with a Pb NIST SRM 981 solution (see Table S4), provided that both the simulated samples and standards had the same matrix composition. This outcome aligns with established evidence requiring matrix matching for the SSB procedure in the absence of an internal standard.^33^

By contrast, matrix modifications induced mass fractionation that could not be corrected by the SSB procedure and was inadequately addressed by the Russell law.^34^ See Figure 3 for the ^204^Pb/^206^Pb IR and Figure S13a,b for the corrections on the ^207^Pb/^206^Pb and ^208^Pb/^206^Pb IRs. To overcome this challenge, we applied the optimized regression model (ORM)^35,36^ in Orbitrap measurement for the first time. The latter was demonstrated to effectively correct for fractionation induced by matrix composition and instrumental factors (instrumental mass bias and its time dependence; see previous references as an example). Details of the applied procedure are provided in the Supporting Information, section “Mass Bias Correction Procedures.” Thallium was used as an internal standard (at the same concentration as Pb, 500 μg/L), and matrix modification was induced by adding high concentrations of Ca (100 μM). Calibration plots for the mass bias correction factors (f) of the three Pb isotopic ratios against the mass bias correction factor of the ^205^Tl/^203^Tl IR are reported in Figure S14. The three models, corresponding to the three Pb IRs, were tested for their predictive performances using a leave-one-out procedure. See Figure 3 for the ^204^Pb/^206^Pb IR and Figure S13a,b for the other two IRs. This procedure ensures optimal accuracy in prediction, with average Pb IRs corrected to within 0.0014% or 14 ppm of the expected value, at worst. Single data points are corrected within 0.4% at worst, with a median of the absolute values of residual mass bias of 0.08%, 0.08%, and 0.1% for the three IRs. The estimation of the linear correlation parameters could be further refined by increasing the number of data points; however, the results presented here serve as robust proof of principle, providing an optimal mass bias correction.

**Figure 3:**
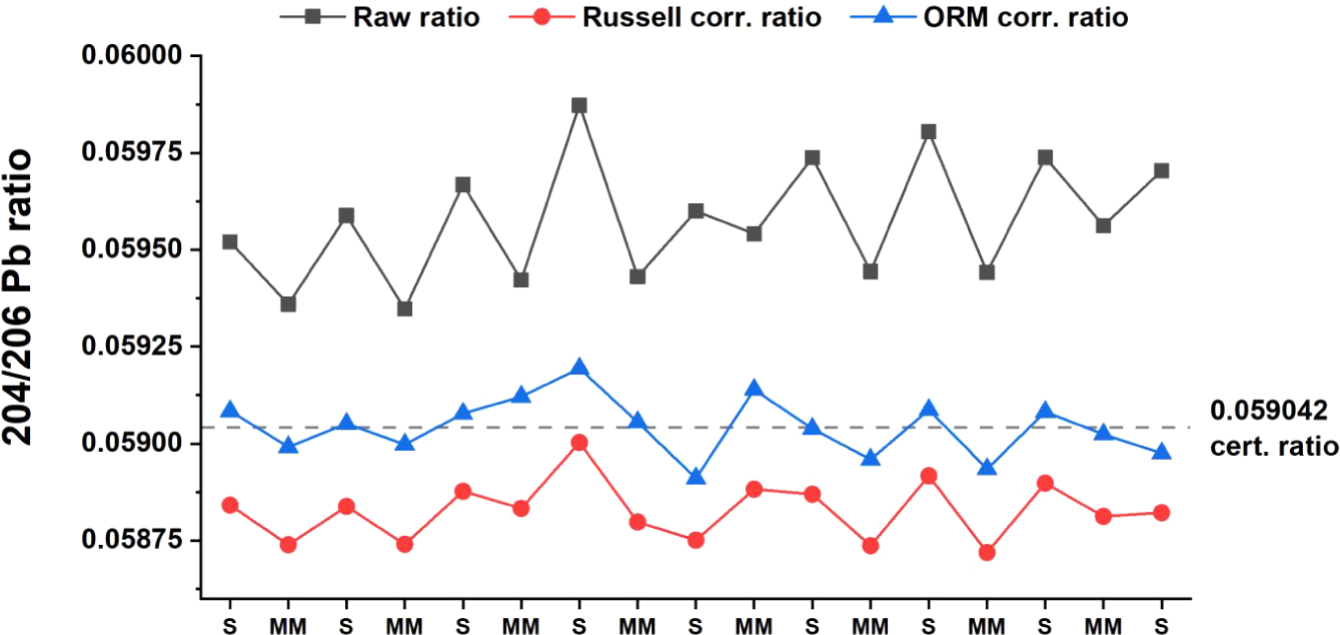
Effect of different mass bias correction methods on the ^204^Pb/^206^Pb isotope ratio. Measures were performed by alternating conventional samples “S” (Pb NIST SRM 981 500 μg/L + Tl 500 μg/L) and matrix-modified samples “MM” (Pb NIST SRM 981 500 μg/L + Tl 500 μg/L + Ca 100 μM). Results were obtained applying different mass bias correction models using thallium as an internal standard. Flow: 15 μL/min. Injected volume: 250 μL. EDTA: 200 μM. Solvent: H_2_O:MeOH 1:1. Buffer: ammonium acetate 5 mM. Resolution: 15k. Microscan: 10. Quad. isol. window: 487–507 *m*/*z*. AGC target: 100%. Max inj. time: 100 ms. HCD: 200%. Scan window: 201.5–209.5 *m*/*z*.

### Precision

Measurement precision is limited by counting statistics, as demonstrated in Figure S15: an acquisition time of 12 min was selected as a compromise between analysis duration and precision. The confidence interval under these short-term experimental conditions is in the 0.021% to 0.093% range when a coverage factor of 2 is used; see Table 2. The long-term precision was also assessed by analyzing 13 replicated measurements of the NIST SRM 981 isotopic standard over approximately 12 h: the data exhibit good stability despite the absence of mass bias correction; see Table 2 and Figure S16. The highest RSD% of the ^204^Pb/^206^Pb ratio is likely due to the low isotopic abundance of ^204^Pb that negatively affects signal reproducibility.

**Table 1:** Qualitative Impact of the Parameters on the Measurement Accuracy and Precision^a^

Parameter	Tested conditions	Effects on mass bias	Effects on precision
**Quadrupole isolation window**	10 amu, 12 amu, **14 amu**, 16 amu	High: optimal width to preserve the isotopic pattern	Low
The isolation width (as an *m*/*z* value) for the precursor ions. The mass range for the peak is centered at the precursor mass and ranges from one-half of the isolation window to either side of the precursor mass.			
**Collision energy**	50%, 75%,100%, 150%, 175%, **200%**	Possible fractionation for low collision energies	Medium: higher energies yield higher S/N
Specifies the collision energy (as a percentage) that the MS uses to accelerate ions into the HCD cell, where fragmentation occurs. Here, HCD 100% ≈ 68 V.			
**Orbitrap scan range**	**5 amu**, 9 amu, 13 amu	Negligible	Negligible
Defines the width of the MS^2^ scan range.			
**AGC Automatic Gain Control**	50%, **100%**, 200%, 500%	Low	Low
Specifies the Automatic Gain Control target. This is a percentage representing the maximum number of charges to accumulate for a given analysis. The normalized base (100%) is 1 × 10^6^.			
**Max. injection time**	1 ms, 10 ms, **100 ms**, 1000 ms	High: shortest times affect low abundance isotopes	High: shortest times reduce the S/N ratio
Specifies the maximum injection time that is allowed to reach the AGC Target. The C-Trap collects ions until it reaches the AGC Target or the max. injection time. The mass spectrometer then transfers the ions to the Orbitrap analyzer.			
**Resolution**	**15000**, 30000, 60000, 120000	Low: unclear trend	Negligible under optimized conditions; may be significant due to longer acquisition times that may lower counting statistics
The mass resolution of the Orbitrap analyzer, which is proportional to 1/sqrt of the mass-to-charge ratio. Mass resolution is defined as the observed *m*/*z* value divided by the smallest mass difference Δ*m*/*z* that can be separated: *m*/Δ*m*. Spectra acquired at higher Orbitrap resolution allow greater resolution in *m*/*z* but take longer to acquire. The selected resolution is specified for a peak at *m*/*z* 200.			
**Microscan number**	1, 2, 5, **10**	Negligible	Medium: better S/N ratio for low abundance isotopes
A microscan is one ion injection followed by ion detection. The MS sums microscans to produce one scan, which improves the S/N ratio of the mass spectral data.			

a The definitions are adapted from the Manuals of the Orbitrap Exploris 120 by Thermo Scientific. S/N: signal-to-noise ratio. Optimal conditions in bold. For further details, see Supporting Information, Section “Effects of Instrumental and Chemical Parameters” and Figures S4–S12.

**Table 2:** Precision of Replicate Measurements of the NIST SRM 981 Solution^a^

NIST SRM 981		^204^Pb/^206^Pb ratio	^207^Pb/^206^Pb ratio	^208^Pb/^206^Pb ratio
**Short-term precision (integration time: 12 min; 16 independent data)**	Median relative confidence interval (cov. fact. = 2) (%)	0.083	0.029	0.023
	Max. – min relative confidence interval (cov. fact. = 2) (%)	0.093–0.076	0.032–0.027	0.028–0.021
**Long-term precision (integration time: 12 min; 13 replicates over 12 h)**	Average	0.059202	0.91523	2.1269
	Std. dev.	0.000063	0.00050	0.00049
	RSD (%)	0.11	0.055	0.023
	Confidence interval (cov. fact. = 2)	0.000035	0.00028	0.00027
	Relative confidence interval (cov. fact. = 2) (%)	0.059	0.030	0.013

a Flow: 15 μL/min. Injected volume: 250 μL. EDTA: 200 μM. Pb NIST 981:500 μg/L. Solvent: H_2_O:MeOH 1:1. Buffer: ammonium acetate 5 mM. Resolution: 15k. Microscan: 10. Quad. isol. window: 490–504 *m/z*. AGC target: 100%. Max. inj. time: 100 ms. HCD cell: 200%. Scan window: 203.5–208.5 *m/z*.

**Table 3:** General and Pb-Specific Instrumental IR Precision Reported in the Literature for Different MS Instrumentations

Instrumentation	RSD%
GDMS	**Generally**: 0.1–1 ^40^
Single Collector ICP-MS	**Generally**: 0.05–0.2 ^41^
Multicollector ICP-MS	**Generally**: down to 0.001 ^42^
	Pb NIST 981:0.004–0.005 ^43^ (Tl as internal standard, exponential law)
TIMS	**Generally**: down to 0.001 ^9^
	Pb NIST 981:0.003–0.012 ^44^ (Pb double spike 204–207)
LS-APGD-Orbitrap	**Generally**: 0.06–0.71 ^21,32,45^
	Pb standard solution: 0.10–0.26 ^21^ (uncorrected IRs)
ESI-Orbitrap (this paper)	Pb NIST 981:0.02–0.1 (Tl as internal standard, ORM approach)

### Detection Capabilities

The procedure was also tested at a Pb total concentration of 100, 10, and 1 μg/L (480 to 4.8 nM) to assess the applicability of the method to low-concentration samples. Precision decreased as Pb concentration declined, especially for the ^204^Pb/^206^Pb ratio, which exhibited the greatest deviation from the expected IR value (see Table S5, data were not mass bias corrected to assess possible fractionation). Achieving precision similar to that reported in Table 2 for a Pb concentration of 500 μg/L at around 100 μg/L would likely require extending the acquisition time, though further reducing the Pb concentration currently seems impractical, especially when aiming to determine the ^204^Pb/^206^Pb concentration with adequate precision.

### Preliminary Comparison with Existing IR Mass Spectrometry Procedures

#### Accuracy

Systematic errors in the determination of IRs in the NIST SRM 981 standard by TIMS and MC-ICP-MS, employing different mass bias correction methods, were compiled and reported in the 3–100 ppm and 10–450 ppm range, respectively, for the two methodologies.^37^ Higher accuracies, in the 2–30 ppm range, were achieved through dedicated procedures in MC-ICP-MS.^38^ Comparable accuracies were also achieved by using the ORM procedure without matrix separation (5–155 ppm).^39^ The present method yielded similar accuracy, with inaccuracies ranging from 2 to 14 ppm, based on an average of 17 measurements, including calcium-spiked NIST 981 standards.

#### Precision

Precision in IR determination is pivotal for attaining reliable isotopic information, provided the mass bias is adequately corrected. Table 3 compares the performance (in terms of RSD%) of the proposed procedure to the ones of existing methods, namely, those based on GD, ICP, or TI ion sources.

As widely recognized, TIMS and MC-ICP-MS offer the highest precision, while measurements by single-collector ICP-MS and glow discharge instruments are associated with higher relative standard deviations. Several experimental conditions (e.g., investigated element and measured ratio, sample matrix vs standard solutions, number of replicates, acquisition time, in addition to the method and frequency of mass bias correction), nonetheless, contribute to determining the overall uncertainties, and fine differences cannot be appreciated. At this early stage of development, the proposed method shows precision between 1and 2 orders of magnitude lower than the best attainable with TIMS and MC-ICP-MS, comparable to those generally achievable with single-collector ICP-MS, and better than the one reported for LS-APGD-Orbitrap. A comprehensive evaluation of the performance in terms of precision, nevertheless, requires extensive implementation of the procedure; see also the Conclusion section.

#### Detection Capabilities

MC-ICP-MS-based procedures offer better detection capabilities, showing uncompromised precision and accuracy down to at least a concentration of 10 μg/L when Pb IRs are determined (see, e.g.,^37^). In contrast, the precision of the present procedure begins to degrade at concentrations 10 times higher.

## Conclusions

We propose a procedure for the determination of isotopic ratios by an unmodified HR-Orbitrap mass spectrometer equipped with a standard ESI source, exploiting the unique selectivity offered by ligand choice, MS^2^ strategy, and detection at ultrahigh resolution. Following the optimization of chemical and mass spectrometric parameters, we demonstrated that Pb IRs can be determined with high accuracy, indicating that existing models for mass bias correction due to sample matrix effects—specifically, the ORM—can also be applied to Orbitrap measurements. With regard to precision, RSD%s in the 0.02–0.1 range were achieved at present; while these figures may suffice for determining isotopic ratios of Pb and other elements, they currently limit the application to elements with low isotopic variability. Notably, the unique selectivity mechanisms, namely ligand choice, MS^2^ filtering, and detection at ultrahigh resolution, are anticipated to shift the burden of achieving interference-free data from time-consuming sample pretreatment to the detection phase. This is particularly relevant in the presence of isobaric interferences, where it may serve as a complementary approach to traditional IR methods, given the previously mentioned limitations in precision.

The procedure is undoubtedly in its early stages, and several aspects and opportunities have yet to be explored. It holds the potential to reliably determine the isotope ratios of any metal ion, although the mass range is limited to *m*/*z* > 40 in the present instrument configuration. Selecting suitable ligand(s) is crucial for expanding the range of analyzable elements as it (they) must enable the formation of a charged complex in the ESI source. The extensive knowledge and diversity of metal complexants can be leveraged for this purpose: variations in ligand charge and chemical properties—such as hard–soft character, binding functional groups, pH sensitivity, and hapticity—offer a wide range of tunable characteristics for optimizing analytical signals. Multiplexing isotopic ratio determination is also theoretically achievable; data shown on the isotopic pattern of the first transition row elements indicate that the procedure may provide isotopic mass spectra of several elements in the same run, although a proof of principle has only been obtained here. The same approach may be applied to multiple groups of elements with similar masses. Multiplexing would be a groundbreaking achievement, moving isotopic data to the multivariate regime and engendering the breadth of knowledge we may attain.

Finally, this study enhances the utility of HR-MS for extracting elemental information. The core strategy—complexation with suitable ligands followed by ligand removal via collisional dissociation—opens new possibilities for applying mass spectrometry to the inorganic domain, with encouraging prospects to advance our understanding of element speciation.

## Supplementary Material



## Notes

Methods, mass bias correction procedures, effect of instrumental and chemical parameters, and additional tables and figures (PDF)

## References

[1] Alexandre, P. Isotopes and the Natural Environment; Springer Textbooks in Earth Sciences, Geography and Environment; Springer: Cham, 2020. DOI: 10.1007/978-3-030-33652-3.

[2] Aggarwal J., Habicht-Mauche J., Juarez C. (2008). Application of Heavy
Stable Isotopes
in Forensic Isotope Geochemistry: A Review. Appl. Geochem..

[3] Zhong Q., Zhou Y., Tsang D. C. W., Liu J., Yang X., Yin M., Wu S., Wang J., Xiao T., Zhang Z. (2020). Cadmium Isotopes
as Tracers in Environmental Studies: A Review. Sci. Total Environ..

[4] Fry, B. Stable Isotope Ecology; Springer: New York, NY, 2006. DOI: 10.1007/0-387-33745-8.

[5] Zhao Y., Zhang B., Chen G., Chen A., Yang S., Ye Z. (2014). Recent Developments
in Application of Stable Isotope Analysis on
Agro-Product Authenticity and Traceability. Food Chem..

[6] Bartelink E. J., Chesson L. A. (2019). Recent Applications of Isotope Analysis to Forensic
Anthropology. Forensic Sci. Re.s.

[7] Coast, G. ; Catahoula, T. ; Counties, G. ; Counties, J. ; County, W. ; County, J. ; County, H. ; Water, T. ; Board, D. ; District, H. C. S. Science for a Changing World; U.S. Geological Survey Circular, 2004.

[8] Komárek M., Ettler V., Chrastný V., Mihaljevič M. (2008). Lead Isotopes
in Environmental Sciences: A Review. Environ.
Int..

[9] Yang L. (2009). Accurate and
Precise Determination of Isotopic Ratios by MC-ICP-MS: A Review. Mass Spectrom. Rev..

[10] von
Blanckenburg F., Oelze M., Schmid D. G., van Zuilen K., Gschwind H.-P., Slade A. J., Stitah S., Kaufmann D., Swart P. (2014). An Iron Stable Isotope Comparison between Human Erythrocytes and
Plasma. Metallomics.

[11] Moens L. J., Vanhaecke F. F., Bandura D. R., Baranov V. I., Tanner S. D. (2001). Elimination
of Isobaric Interferences in ICP-MS, Using Ion–Molecule Reaction
Chemistry: Rb/Sr Age Determination of Magmatic Rocks, a Case Study. J. Anal. At. Spectrom..

[12] Bandura D. R., Baranov V. I., Litherland A. E., Tanner S. D. (2006). Gas-Phase Ion–Molecule
Reactions for Resolution of Atomic Isobars: AMS and ICP-MS Perspectives. Int. J. Mass Spectrom..

[13] Hu Q., Noll R. J., Li H., Makarov A., Hardman M., Cooks R. G. (2005). The Orbitrap: A New Mass Spectrometer. J. Mass Spectrom..

[14] Zubarev R. A., Makarov A. (2013). Orbitrap Mass Spectrometry. Anal.
Chem..

[15] Neubauer C., Kantnerová K., Lamothe A., Savarino J., Hilkert A., Juchelka D., Hinrichs K. U., Elvert M., Heuer V., Elsner M., Bakkour R., Julien M., Öztoprak M., Schouten S., Hattori S., Dittmar T. (2023). Discovering Nature’s
Fingerprints: Isotope Ratio Analysis on Bioanalytical Mass Spectrometers. J. Am. Soc. Mass Spectrom..

[16] Kantnerová K., Kuhlbusch N., Juchelka D., Hilkert A., Kopf S., Neubauer C. (2024). A Guide to
Precise Measurements of Isotope Abundance
by ESI-Orbitrap MS. Nat. Protoc..

[17] Neubauer C., Crémière A., Wang X. T., Thiagarajan N., Sessions A. L., Adkins J. F., Dalleska N. F., Turchyn A. V., Clegg J. A., Moradian A., Sweredoski M. J., Garbis S. D., Eiler J. M. (2020). Stable Isotope Analysis
of Intact
Oxyanions Using Electrospray Quadrupole-Orbitrap Mass Spectrometry. Anal. Chem..

[18] Hilkert A., Böhlke J. K., Mroczkowski S. J., Fort K. L., Aizikov K., Wang X. T., Kopf S. H., Neubauer C. (2021). Exploring the Potential
of Electrospray-Orbitrap for Stable Isotope Analysis Using Nitrate
as a Model. Anal. Chem..

[19] Agnes G. R., Horlick G. (1995). Effect of Operating
Parameters on Analyte Signals in
Elemental Electrospray Mass Spectrometry. Appl.
Spectrosc..

[20] Marcus R. K., Quarles C. D., Barinaga C. J., Carado A. J., Koppenaal D. W. (2011). Liquid
Sampling-Atmospheric Pressure Glow Discharge Ionization Source for
Elemental Mass Spectrometry. Anal. Chem..

[21] Williams T.
J., Hoegg E. D., Bills J. R., Marcus R. K. (2021). Roles of Collisional
Dissociation Modalities on Spectral Composition and Isotope Ratio
Measurement Performance of the Liquid Sampling – Atmospheric
Pressure Glow Discharge/Orbitrap Mass Spectrometer Coupling. Int. J. Mass Spectrom..

[22] Agnes G. R., Horlick G. (1992). Electrospray Mass Spectrometry as a Technique for Elemental
Analysis: Preliminary Results. Appl. Spectrosc..

[23] Stewart I. I. (1999). Electrospray
Mass Spectrometry: A Tool for Elemental Speciation. Spectrochim. Acta Part B At. Spectrosc..

[24] Baron D., Hering J. G. (1998). Analysis of Metal-EDTA
Complexes by Electrospray Mass
Spectrometry. J. Environ. Qual..

[25] Beck S. (2021). Fragmentation
Behavior of EDTA Complexes under Different Activation Conditions. J. Mass Spectrom..

[26] Tsednee M., Huang Y. C., Chen Y. R., Yeh K. C. (2016). Identification of
Metal Species by ESI-MS/MS through Release of Free Metals from the
Corresponding Metal-Ligand Complexes. Sci. Rep..

[27] Blanchard E., Paredes E., Rincel A., Nonell A., Chartier F., Bresson C. (2021). Determining
the Isotopic Composition of Elements from
the Electrospray Ionization Mass Spectra of Their Chemical Species. J. Anal. At. Spectrom..

[28] Catanzaro E. J., Murphy T. J., Shields W. R., Garner E. L. (1968). Absolute Isotopic
Abundance Ratios of Common, Equal-Atom, and Radiogenic Lead Isotopic
Standards. J. Res. Natl. Bur. Stand. Sect. A
Phys. Chem..

[29] Cheema A. I., Liu G., Yousaf B., Abbas Q., Zhou H. (2020). A Comprehensive Review
of Biogeochemical Distribution and Fractionation of Lead Isotopes
for Source Tracing in Distinct Interactive Environmental Compartments. Sci. Total Environ..

[30] Zhu X.-K., Benefield J., Coplen T. B., Gao Z., Holden N. E. (2021). Variation
of Lead Isotopic Composition and Atomic Weight in Terrestrial Materials
(IUPAC Technical Report). Pure Appl. Chem..

[31] Eiler J., Cesar J., Chimiak L., Dallas B., Grice K., Griep-Raming J., Juchelka D., Kitchen N., Lloyd M., Makarov A., Robins R., Schwieters J. (2017). Analysis of
Molecular Isotopic Structures at High Precision and Accuracy by Orbitrap
Mass Spectrometry. Int. J. Mass Spectrom..

[32] Shrestha S., Goodwin J. V., Manard B. T., Marcus R. K. (2025). Parametric Optimization
of the Liquid Sampling-Atmospheric Pressure Glow Discharge Ionization
Source Coupled to an Orbitrap Mass Spectrometer for Neodymium Isotope
Ratio Determinations. Int. J. Mass Spectrom..

[33] Mason T. F. D., Weiss D. J., Horstwood M., Parrish R. R., Russell S. S., Mullane E., Coles B. J. (2004). High-Precision
Cu and Zn Isotope
Analysis by Plasma Source Mass Spectrometry. J. Anal. At. Spectrom..

[34] Russell W. A., Papanastassiou D. A., Tombrello T. A. (1978). Ca Isotope Fractionation on the Earth
and Other Solar System Materials. Geochim. Cosmochim.
Acta.

[35] Karasiński J., Tupys A., Yang L., Mester Z., Halicz L., Bulska E. (2020). Novel Approach for the Accurate Determination
of Se
Isotope Ratio by Multicollector ICP-MS. Anal.
Chem..

[36] Zhu Z., Meija J., Tong S., Zheng A., Zhou L., Yang L. (2018). Determination of the Isotopic Composition of Osmium Using MC-ICPMS. Anal. Chem..

[37] Gallon C., Aggarwal J., Flegal A. R. (2008). Comparison
of Mass Discrimination
Correction Methods and Sample Introduction Systems for the Determination
of Lead Isotopic Composition Using a Multicollector Inductively Coupled
Plasma Mass Spectrometer. Anal. Chem..

[38] Ortega G.
S., Pécheyran C., Bérail S., Donard O. F. X. (2012). A Fit-for Purpose
Procedure for Lead Isotopic Ratio Determination in Crude Oil, Asphaltene
and Kerogen Samples by MC-ICPMS. J. Anal. At.
Spectrom..

[39] Karasiński J., Bulska E., Halicz L., Tupys A., Wagner B. (2023). Precise Determination
of Lead Isotope Ratios by MC-ICP-MS without Matrix Separation Exemplified
by Unique Samples of Diverse Origin and History. J. Anal. At. Spectrom..

[40] Heumann K. G., Gallus S. M., Rädlinger G., Vogl J. (1998). Precision and Accuracy
in Isotope Ratio Measurements by Plasma Source Mass Spectrometry. J. Anal. At. Spectrom..

[41] Penanes P. A., Galán A. R., Huelga-Suarez G., Rodríguez-Castrillón J. Á., Moldovan M., Garcia Alonso J. I. (2022). Isotopic Measurements Using ICP-MS:
A Tutorial Review. J. Anal. At. Spectrom..

[42] Van
Acker T., Theiner S., Bolea-Fernandez E., Vanhaecke F., Koellensperger G. (2023). Inductively Coupled Plasma Mass Spectrometry. Nat. Rev. Methods Primers.

[43] Ishida M., Fujinaga K., Tanimizu M., Ishikawa T., Nagaishi K., Kato Y. (2024). New Pb Isotopic Data
from Japanese Hydrothermal Deposits for Tracing
Heavy Metal Sources. Geochemistry.

[44] Taylor R. N., Ishizuka O., Michalik A., Milton J. A., Croudace I. W. (2015). Evaluating
the Precision of Pb Isotope Measurement by Mass Spectrometry. J. Anal. At. Spectrom..

[45] Hoegg E. D., Godin S., Szpunar J., Lobinski R., Koppenaal D. W., Marcus R. K. (2021). Resolving Severe
Elemental Isobaric Interferences with
a Combined Atomic and Molecular Ionization Source-Orbitrap Mass Spectrometry
Approach: The 87Sr and 87Rb Geochronology Pair. Anal. Chem..

