# Supplementary material for: Metal Ion Isotope Ratio Using ESI-Orbitrap HRMS: Proof of Concept and Initial Performance Evaluation for Lead Isotopic Ratios
Source: Anal Chem. 2025 Jun 17;97(25):13176–83. doi: 10.1021/acs.analchem.5c01033 (PMC12224156; doi:10.1021/acs.analchem.5c01033)
Supplement: Supplementary file 1 [file ac5c01033_si_001.pdf]

SUPPORTING INFORMATION

Metal Ion Isotope Ratio Using ESI-Orbitrap HRMS: proof of concept and initial performance evaluation for lead isotopic ratios

Gianluca Roncoroni[a], Davide Spanu[a], Gilberto Binda[b], and Damiano Monticelli[a] *

[a] Università degli Studi dell'Insubria, Dipartimento di Scienza e Alta Tecnologia, via Valleggio 11, 22100 Como, Italy.

[b] Norwegian Institute for Water Research, Økernveien 94, 0349 Oslo, Norway.

*corresponding author: damiano.monticelli@uninsubria.it

Contents

1. **Methods** S-2
   - 1.1 **Reagents, solutions and standards** S-2
   - 1.2 **Instrumentation** S-3
   - 1.3 **Data acquisition and processing** S-3
2. **Mass bias correction procedures** S-4
   - 2.1 **Standard-sample bracketing (SSB)** S-4
   - 2.2 **Russell law** S-4
   - 2.3 **Optimized regression model (ORM)** S-4
3. **Effects of instrumental and chemical parameters** S-6
4. **Tables** S-8
5. **Figures S1-S16** S-11
6. **References of SI** S-23

1. Methods

1.1 Reagents, solutions and standards

All the solution and sample containers were cleaned before use by soaking in a detergent solution (Nalgene L900, 4 mL/L) for 2 days, rinsing with ultrapure water and finally soaking in a 2% HNO_3 solution for 2 days. Stock solutions were prepared in HDPE 30 mL bottles (Nalgene), and sample solutions for Orbitrap™ analysis were prepared in 5 mL PP microcentrifuge tubes (VWR) and then transferred in HPLC 1.5 mL glass vial (Thermo Scientific™). To prevent environmental contamination of standards and samples, their handling and preparation were carried out under a laminar flow hood (BIOAIR aura HZ 72T).

Ultrapure water (resistivity 18.2 MΩcm) was produced by a Sartorius Arium Mini Plus system, whereas ultrapure HNO_3 and HCl were produced by a Milestone DuoPUR Subboiling distillation system [1], starting respectively from HNO_3 65% for analysis-ISO (Carlo Erba), and HCl ≥37%, puriss. p.a. (Sigma-Aldrich). 10 mM ammonium acetate buffer was prepared in ultrapure water mixing adequate amounts of ammonium hydroxide solution (TraceSELECT Ultra, for trace analysis, NH_3 ≥25% in H_2O, Fluka) and acetic acid (puriss. p.a., Fluka). LC/MS grade methanol (Carlo Erba) and a 0.1% solution of formic acid (99% formic acid, LC/MS grade, Carlo Erba) in ultrapure water were used as HPLC solvents to transfer the sample to the ESI source.

Ethylenediaminetetraacetic acid (EDTA) extra pure ≥98% (MERCK) was used as the chelating agent and purified before use by precipitation from an aqueous concentrated solution using ultrapure concentrated HCl. Precipitate was rinsed with small aliquots of ultrapure water, dried under vacuum, and then used to prepare a 5 mM stock solution in ultrapure water. Small volumes of an ammonium hydroxide solution were added to increase EDTA solubility whenever needed.

An acidic solution of NIST Standard Reference Material (SRM) 981 isotopic standard was prepared dissolving an opportune amount of standard material in 2 mL of a 1:1 mixture of ultrapure HNO_3 and ultrapure water. Dissolution was accelerated by gentle heating, and the solution was then diluted to the final concentration of 500 mg/kg with ultrapure water. An acidic solution of NIST SRM 982, prepared by the above-described procedure, was used to validate the method.

A multi-standard solution containing 10 mg/kg of Cr, Fe, Ni, Cu, Zn, Ga, In, and Pb was used to explore the applicability of the proposed method. This solution was prepared by diluting single elemental standards of each element in ultrapure water. Standards were purchased from Fluka Analytical (ICP standards, 1000 mg/L, single element standard: Fe, Ni, Cu, Zn, Ga, and Pb), from Merck (ICP standard, 1000 mg/L: In) and from Czech Metrology Institute (Aqueous calibration solution, 1000 mg/L: Cr in +III oxidation state to enable complexation by EDTA). An IC standard containing 1000 mg/L of Ca^{2+} (TraceCERT, Sigma-Aldrich) was used to simulate an environmental matrix with a final calcium concentration of 100 µM. Thallium in +III oxidation state, used as internal standard for some validation measures, was prepared from thallium(III) nitrate trihydrate (Sigma-Aldrich).

The solutions to be analyzed were prepared by adding adequate volumes of the EDTA, 5 mM stock solution, and Pb solutions (NIST 981 or 982) to a 1:1 mixture of LC/MS grade MeOH and 10 mM aqueous ammonium acetate buffer at pH 4.30. Testing solutions for unbuffered conditions were prepared in a 1:1 mixture of LC/MS grade MeOH and ultrapure water containing formic aci d 0.1%.

The best results were achieved using an EDTA concentration of 200 µM, a Pb concentration of 500 µg/L, and 5 mM ammonium acetate buffer (pH 4.30) in a 1:1 MeOH:H_2O mixture.

1.2 Instrumentation

Optimized LC, ion source, and mass scan parameters are reported in Table S1: ion source voltage and gas flows were optimized using an auto-tune tool available in TUNE™ software (Thermo Scientific™) to maximize the intensity of the ^{208}Pb-ligand precursor ions.

The modification of ion transfer tube temperature (220-340°C), vaporizer temperature (40-150°C), and radiofrequency voltage (30-100%) values in the reported ranges did not result in perceptible variations in signal intensity and mass bias: their values have not been altered from the default settings.

Preliminary acquisitions were executed via direct infusion mode with a sample flow of 5 µL/min, whereas all subsequent analyses used the autosampler for solution introduction into the ESI probe. A "washout flow" of 300 µL/min was introduced in the latter case to facilitate line cleaning between samples and to reduce the overall analysis time, see Figure S1 for flow rate settings and signal trend during the analysis.

A portable pHmeter HI 9125 (Hanna) and a combined glass electrode (AMEL Electrochemistry) were used for pH measurements.

1.3 Data acquisition and processing

The preliminary measurements via direct infusion were conducted under manual control using the TUNE™ software (Thermo Scientific™), while those via LC-MS were performed using the Xcalibur™ software (Thermo Scientific™). Evaluation of preliminary data (e.g., feasibility study, peak analysis, and recognition) was carried out using the Freestyle™ software (Thermo Scientific™). Extraction of the chromatograms necessary for isotopic analyses was accomplished using the QualBrowser software (Thermo Scientific™).

Further data manipulation was executed using an in-house Microsoft Excel worksheet. Extracted Ion Chromatograms (time vs intensity) for the isotopes of interest were limited to the timeframe of sample introduction, from min 3 to min 15, see Figure S1. Data were subsequently filtered to remove, if present, intensity values equal to zero, or outside an interval of ± 3 standard deviations. Filtered data were then used to calculate the IRs (normalized on ^{206}Pb isotope), and the result was expressed as average ± 1 standard deviation of these values.

2. Mass bias correction procedures

The Standard-Sample Bracketing (SSB), Russell law and Optimized Regression Model (ORM) approaches were tested during the validation phase. For other details about correction procedures see [2,3]

2.1 Standard-sample bracketing (SSB)

The standard-sample bracketing is based on sequential measurements of standards of known isotopic composition and unknown samples. For a couple of two isotopes i and j, the isotope correction factors ($k_{std,i/j}$) are first calculated by dividing the certified isotopic ratio $R_{std,i/j}$ by the measured isotopic ratio $r_{std.meas.,i/j}$ (1) in two adjacent standards that bracket the sample. The corrected ratio of the analyte in the sample, $R_{samp.,i/j}$, is calculated by multiplying the average $K_{std,i/j}$ of the two $k_{std,i/j}$ values by the measured isotope ratio $r_{samp.,i/j}$ (2). Equations are reported from [2].

(1) $$k_{std,i/j} = R_{std,i/j}/r_{std.meas.,i/j}$$

(2) $$R_{samp.,i/j} = K_{std,i/j} * r_{samp.meas.\ i/j}$$

2.2 Russell law

The Russell law is a simple correction model which assumes that a correction factor f, obtained from a single isotope ratio allows the correction of the measured IRs for any other isotope pair. In this paper, thallium was used as the internal standard, assuming a natural isotope ratio $^{205}Tl/^{203}Tl$ of 2.3871, as recommended by [4], and its correction factor was used to correct Pb isotope ratios, following (3).

(3) $$R_{i/j} = r_{i/j} * \left(\frac{mass_i}{mass_j}\right)^f$$

2.3 Optimized regression model (ORM)

The fundamental concept is to link the isotope ratio of the element being analyzed to one or more isotope ratios in the isotopic reference material. The optimized regression method requires a primary isotopic reference material from another element. The calibrant can consist of either a pair of isotopes from the analyte element itself or isotopes of a different element with a known isotopic composition introduced into the sample. In our case, thallium is used as internal standard.

The ORM uses the thallium fractionation coefficient $f_{205/203}$ to obtain more appropriate Pb correction factors ($f_{i/j}$) to calculate Pb IRs (and not directly used to correct Pb IRs as done in the Russell law). A general $f_{i/j}$ is calculated as reported in (4).

(4) $$f_{i/j} = \log\left(\frac{R_{theo.,i/j}}{r_{meas.,i/j}}\right) / \log\left(\frac{mass_i}{mass_j}\right)$$

Calibration is essential to establish a mathematical relationship between the correction factors of the internal standard, Tl, and the analyte, Pb. This process was performed using Pb NIST SRM 981 at a concentration of 500 µg/L, along with a solution of natural Tl^{3+} at the same concentration. By plotting the correction factor ($f_{205/203}$) of the $^{205}Tl/^{203}Tl$ ratio against each fractionation coefficient of the Pb ratios, namely $^{204}Pb/^{206}Pb$, $^{207}Pb/^{206}Pb$ and $^{208}Pb/^{206}Pb$, a mathematical relationship between them was determined using a least-squares approach. During the analysis of unknown samples, Tl serves as a proxy to recalculate the correction factors ($f_{204/206}$, $f_{207/206}$, $f_{208/206}$) for each Pb isotope ratio. Once the appropriate Pb correction factor is determined, the corrected isotope ratio is calculated using (5).

(5) $$R_{corr.,i/j} = r_{meas.,i/j} * \left(\frac{mass_i}{mass_j}\right)^f$$

In this study, 17 standards of NIST SRM 981, 500 µg/L, including 8 with matrix modification were measured (addition of Ca 100 µM). To validate the model a leave-one-out approach was employed: 16 samples were used to calibrate the method and the regression equation was used to recalculate the measure excluded from the calibration set.

3. Effects of instrumental and chemical parameters

See Table 1 for definitions of investigated parameters.

Initially, the effect of the quadrupole isolation window was assessed (see Figure S4 and Figure S5a and STEP 4 in the section Method outline on the main text). The apparently wider than needed quadrupole selection window, spanning 14 amu, is due to the bell-shaped [5] (not rectangular) quadrupole transmission window. The latter requires a larger isolation window to avoid incomplete transmission at the window edges, as observed by Williams et al [6].

Secondly, investigating the impact of collision energy showed that maximum levels yielded the highest precision with the lowest mass fractionation (Figure S5b and effect on Pb signal intensity in Figure S6). The precision improves because of enhanced signal intensity in MS^2, driven by increased complex dissociation (Figure S6): the mechanism behind fractionation as a function of collision energy in MS^2 is conversely at present unclear.

On the other hand, the Orbitrap scan range did not affect the studied parameters and may be limited to the smallest interval needed to detect the isotopes under investigation (Figure S7).

As mentioned in STEP 4 of the Method Outline section, two parameters govern ion accumulation in the C-Trap: the Automatic Gain Control™ (AGC) target value and the maximum injection time. Accordingly, the influence of these two parameters was investigated. Ion accumulation in the C-Trap occurs until one of the following two conditions is met: either the preset number of ions is collected (resulting in a well-defined packet size, determined by the AGC target value) or the maximum injection time is reached (in which case the accumulation of ions stops regardless of the ion packet size in the C-Trap). Since these two parameters simultaneously control the filling of the C-Trap, the effect of each parameter was studied independently, by maximizing the value of the non-target parameter. For example, to isolate the impact of the ion packet size (controlled by the AGC target value), the maximum injection time was set to its highest possible value (1000 ms). This approach ensures that ion accumulation and transmission are governed by the AGC target value, as under our measurement conditions, the proper number of ions is collected in less time than the maximum injection time of 1000 ms. The same principle was applied when studying the effect of the maximum injection time. Results are reported in Figure S8a and Figure S8b. The AGC target value showed a negligible effect, at least within the investigated range of 50–200%, since the resulting ion accumulation provided a sufficient S/N ratio even for the lowest-abundance isotope, ^{204}Pb. However, the lowest accumulation times, 1 ms and 10 ms, led to degraded precision, particularly for the ^{204}Pb signal, impacting both the precision and accuracy of its IR. The optimal values for these parameters are reported in Table 1, and were applied in subsequent experiments.

Next, the effect of the solution composition on Pb signal intensity in MS^2 and IR determination was evaluated. The results (Figure S9) indicate that the H_2O:MeOH 1:1 mixture, initially selected based on literature references [7], maximized analyte signals. Interestingly, MeOH performed better than MeCN, and higher aqueous content negatively affected the signal for both the tested organic solvents: the 1:1 H_2O:MeOH solution was selected, ensuring good sensitivity and compatibility with water-based samples. The sensitivity is anticipated to increase further in a 100% organic solvent. However, a solvent exchange step would be necessary for samples that are typically water-based.

The use of a pH buffer, specifically 5 mM ammonium acetate at pH 4.30, was also investigated to provide a selectivity mechanism for complex formation and to enable the matching of the standard and sample matrix, the latter in agreement with the principle of equal treatment [8]. Data evidenced a matrix-induced modification in the IRs (compare Figures S5, S7, S8 with no added buffer, and Figures S10, S11, S12 for buffered solutions) and a slight improvement in the precision, the latter possibly caused by a better signal stability (see the

paragraph Accuracy under the Validation section for mass bias correction). The ammonium acetate buffer at pH 4.30 was accordingly adopted for all the subsequent measurements.

The effect of the eluent flow rate was also assessed, though, as expected, it did not show appreciable effects prompting the use of the previously used flow rate of 15 µL/min to minimize sample and eluent consumption (see Figure S10).

Subsequently, different ligand concentrations were tested (Figure S11). As expected, no clear difference emerged when the EDTA concentration was changed in the range of 50-400 µM: an intermediate concentration of 200 µM was selected to ensure Pb complexation in sample matrices, even in the presence of other metal ions that may interfere with EDTA ability to bind Pb.

Finally, the effect of resolution (Figure S12a) and of the number of microscans (Figure S12b) were investigated. Resolution modifies the mass bias, possibly due to the longer residence time of the ions in the mass analyzer when a higher resolution is required. The longer residence time may also reduce IR precision by limiting the number of data points; however, under optimized conditions (12-minute acquisition time), precision remains unaffected (Figure S12a) though the number of data points halved when the resolution was set to its maximum value of 120k.

The precision of the measurements is apparently determined by the number of microscans (Figure S12b): nevertheless, the parameter controlling precision is the total acquisition time as reported in Figure S 15, and changing the number of microscans affects only the timeframe of signal averaging. The adopted acquisition time of 12 minutes seems to be a good compromise between analysis time and achievable precision.

The effects of the investigated parameters on mass bias and precision are summarized in Table 1 and a typical Pb isotope mass spectrum under optimized conditions is reported in Figure S3.

4. Tables

LC parameters	
Analysis time (min)	25
Eluent mixture	Isocratic, MeOH:H_2O(0.1% formic acid) 1:1
Pump flow (µL/min)	0-15 min: 15<br>15-25 min (washing time): 300
Injected volume (µL)	250
**Ion source parameters**	
Ion spray voltage (V)	4600
Sheath gas (arb)	5
Aux gas (arb)	4
Sweep gas (arb)	0
Ion transfer tube temp. (°C)	320
Vaporizer temp. (°C)	40
**Scan parameters**	
Scan type	MS^2
Isolation window (m/z)	Pb only: 490-504
	Pb and Tl: 487-507
Collision energy type	Normalized
HCD collision energy (%)	200
Orbitrap resolution	15000
Scan range (m/z)	Pb only: 203.5-208.5
	Pb and Tl: 201.5-209.5
RF lens (%)	70
AGC™ target (%)	100
Max injection time (ms)	100
Microscan number	10
Source fragmentation	Off
EASY-IC	Off

Table S1. Optimized parameter used for Pb-EDTA LC-Orbitrap analysis.

Element	Isotope	Accurate mass	Mass delta (ppm)	Meas. ratio	Natural ratio	Error on isotope ratio (%)	Natural abundance (%)[1]
Cr	50	49.9454	-1.29	0.033467	0.051856	-35.5	4.345
	52	51.9399	-1.05	-	-	-	83.789
	53	52.9400	-1.24	0.098819	0.113392	-12.9	9.501
	54	53.9383	-0.99	0.023194	0.028226	-17.8	2.365
Fe	54	53.9390	-1.45	0.044571	0.063703	-30.0	5.845
	56	55.9343	-1.47	-	-	-	91.754
	57	56.9348	-1.35	0.019079	0.023094	-17.4	2.119
	58	57.9328	0.32	0.002826	0.003073	-8.0	0.282
Ni	58	57.9347	-1.67	-	-	-	68.0769
	60	59.9301	-1.69	0.395945	0.385198	2.8	26.2231
	61	60.9304	-1.53	0.015458	0.016744	-7.7	1.1399
	62	61.9277	-1.27	0.056869	0.053388	6.5	3.6345
	64	63.9273	-1.14	0.013448	0.013596	-1.1	0.9256
Cu	63	62.9290	-1.29	-	-	-	69.17
	65	64.9272	-1.02	0.442906	0.445713	-0.6	30.83
Zn	64	63.9285	-1.28	-	-	-	48.63
	66	65.9254	-1.07	0.441199	0.57372	-23.1	27.9
	67	66.9265	-0.82	0.011375	0.08431	-86.5	4.1
	68	67.9242	-0.88	0.281818	0.385564	-26.9	18.75
	70	ND	ND	ND	0.012749	-	0.62
Ga	69	68.9250	-0.84	-	-	-	60.108
	71	70.9241	-0.92	0.658326	0.663672	-0.8	39.892
In	113	112.9034	-0.67	0.039682	0.044823	-11.5	4.29
	115	114.9032	-1.44	-	-	-	95.71
Pb	204	203.9726	0.95	0.028363	0.026717	6.2	1.4
	206	205.9741	1.76	0.472328	0.459924	2.7	24.1
	207	206.9757	0.84	0.438141	0.421756	3.9	22.1
	208	207.9763	0.66	-	-	-	52.4

Table S2. Elements detected in MS^2 using the proposed procedure (EDTA as ligand). Measured and natural isotope ratios are calculated on the most abundant isotope. [1]: data from IUPAC Commission on Isotopic Abundances and Atomic Weights online database (www.ciaaw.org). Measurement duration: 10 min. Measurement conditions: EDTA: 200 µM. Direct injection flow: 5 µL/min. Solvent: H_2O:MeOH 1:1. pH: 4.20. Resolution: 120k. Microscan: 10. Quad. Isolation window: 338-368 m/z (Cr, Fe, Ni, Cu, Zn, Ga), 397-407 m/z (In), 490-502 (Pb). AGC target: 10^6 charges (Cr, Fe, Ni, Cu, Zn, Ga; In); 10^5 charges (Pb). Max. injection time: 100 ms. Injection control: AGC (Cr, Fe, Ni, Cu, Zn, Ga; In); time (Pb). HCD: 200%. Scan window: 48-71.5 m/z (Cr, Fe, Ni, Cu, Zn, Ga), 112-117 m/z (In), 203.5-208.5 m/z (Pb).

Accurate mass (m/z)	Formula	Theoretical mass (m/z)	Delta (ppm)
293.0981	$C_{10}H_{17}N_2O_8$	293.09794	0.55
499.0603	$C_{10}H_{15}N_2O_8{}^{208}Pb$	499.05893	2.81
498.0503	$C_{10}H_{15}N_2O_8{}^{207}Pb$	489.05817	2.21
497.0576	$C_{10}H_{15}N_2O_8{}^{206}Pb$	497.05674	1.69
495.0569	$C_{10}H_{15}N_2O_8{}^{204}Pb$	495.05532	3.20

Table S3. EDTA and Pb-EDTA peaks analysis results. See also Figure S2.

Sample	N = 10	$^{204}Pb/^{206}Pb$ ratio	$^{207}Pb/^{206}Pb$ ratio	$^{208}Pb/^{206}Pb$ ratio
NIST SRM 981 [a]	**Measured ratio**	0.059051	0.91485	2.1680
	**Cert. ratio**	0.059042	0.91464	2.1681
	**Difference %**	0.015	0.023	-0.004
Mixture of NIST SRM 981 and NIST SRM 982 [b]	**Measured ratio**	0.058769	0.91117	2.1595
	**Expected ratio**	0.058785	0.91104	2.1587
	**Difference %**	-0.026	0.015	0.037

*Table S4. Accuracy correction using the Standard-Sample Bracketing approach, using NIST SRM 981 as correction standard. 10 samples were corrected using 11 standards. Difference % is calculated as ($R_{meas.}$-$R_{expect.}$)/$R_{expect.}$*100. Flow: 15 µL/min. Injected volume: 250 µL. EDTA: 200 µM. Pb NIST 981: 500 µg/L. Pb NIST 982: 5 µg/L. Solvent: H_2O:MeOH 1:1. Buffer: ammonium acetate 5 mM. Resolution: 15k. Microscan: 10. Quad. isol. window: 490-504 m/z. AGC target: 100%. Max inj. time: 100 ms. HCD: 200%. Scan window: 203.5-208.5 m/z. [a] NIST SRM 981 is used to correct NIST SRM 981 itself. [b] A solution containing a mixture of NIST SRM 981 (99.514%) and 982 (0.48640%) was prepared by weighting to induce a small difference in the isotopic ratios of NIST SRM 981, approximately - 4‰ in isotopic delta for $^{204}Pb/^{206}Pb$, $^{207}Pb/^{206}Pb$ and $^{208}Pb/^{206}Pb$ isotope ratios. Pure NIST SRM 981 is used to correct isotope ratios of the mixture.*

Pb conc (µg/L)		$^{204}Pb/^{206}Pb$ ratio	$^{207}Pb/^{206}Pb$ ratio	$^{208}Pb/^{206}Pb$ ratio
1	**Measured ratio**	0.046522	0.89270	2.1232
	**SD of the avg**	0.000407	0.000649	0.001236
	**RSD%**	1.78	0.15	0.12
	**Difference %**	-21.20	-2.40	-2.07
10	**Measured ratio**	0.05110	0.903935	2.1335
	**SD of the avg**	0.000342	0.000629	0.001215
	**RSD%**	1.34	0.14	0.11
	**Difference %**	-13.45	-1.17	-1.60
100	**Measured ratio**	0.058555	0.917161	2.1390
	**SD of the avg**	0.000049	0.000241	0.000482
	**RSD%**	0.17	0.05	0.05
	**Difference %**	-0.83	0.28	-1.34

Table S5. Effect of Pb concentration on precision and accuracy of Pb IRs. Data not corrected for mass bias. Flow: 15 µL/min. Injected volume: 250 µL. EDTA: 200 µM. Pb NIST 981: 500 µg/L. Solvent: H_2O:MeOH 1:1. Buffer: ammonium acetate 5 mM. Resolution: 15k. Microscan: 10. Quad. isol. window: 490-504 m/z. AGC target: 100%. Max inj. time: 100 ms. HCD: 200%. Scan window: 203.5-208.5 m/z.

5. Figures S1-S16

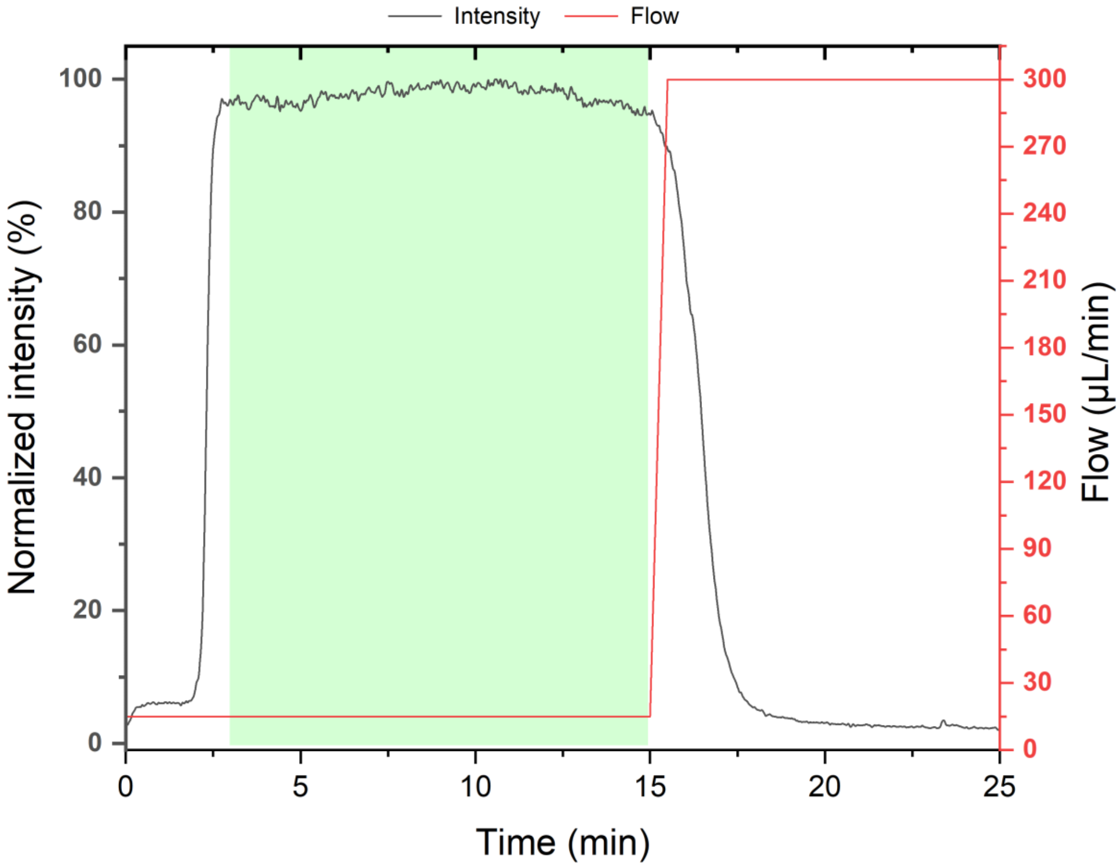

Figure S1. Typical "chromatogram" and flow program. For an injected volume of 250 µL and a 15 µL/min flow rate, a signal of 12 minutes is obtained (highlighted in green). The presence of 5 mM ammonium acetate buffer improves signal stability and reproducibility. Moreover it facilitates sample preparation procedure.

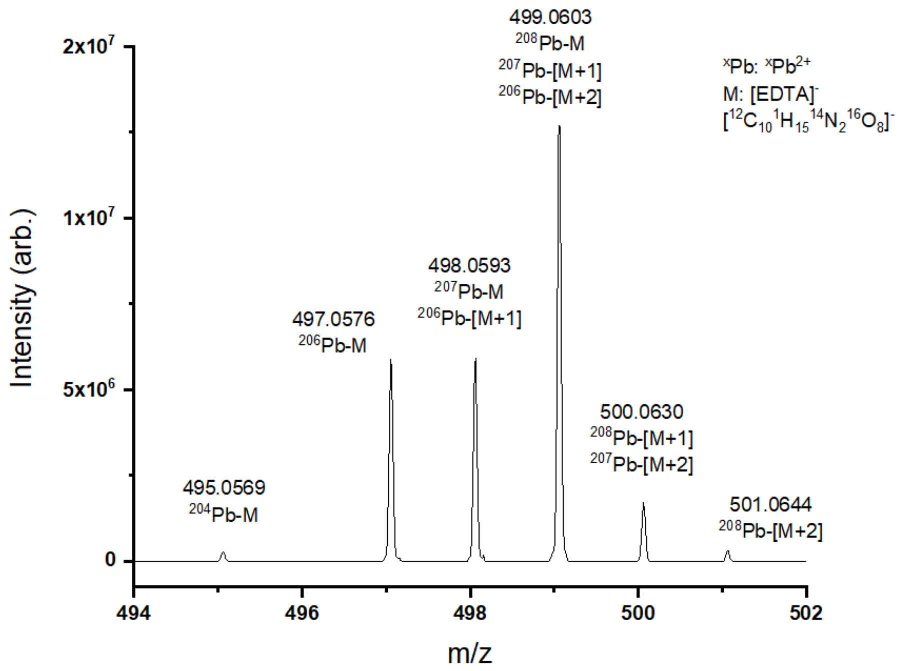

Figure S2. Pb-EDTA MS[1] spectrum. See also Table S2.

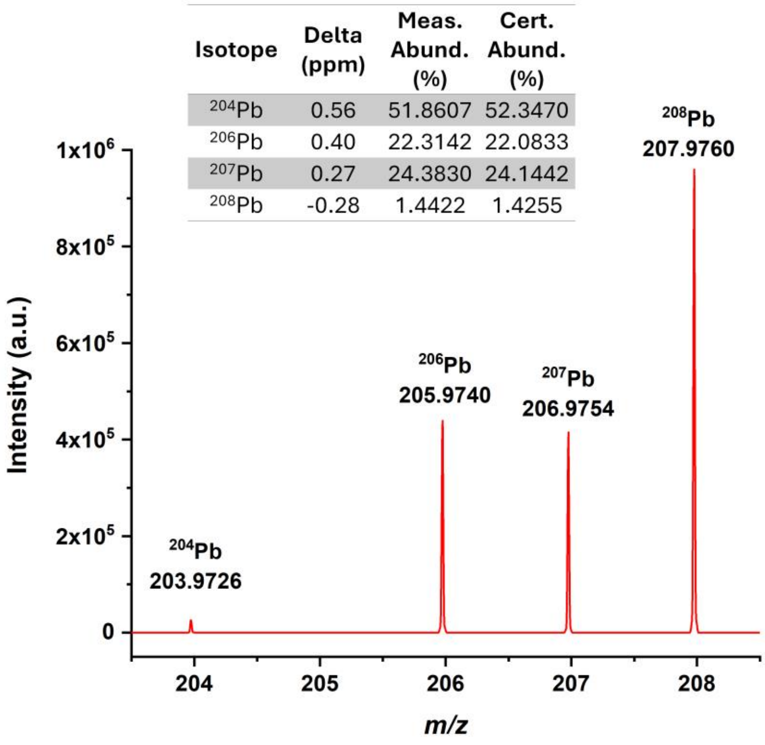

Isotope	Delta (ppm)	Meas. Abund. (%)	Cert. Abund. (%)
^{204}Pb	0.56	51.8607	52.3470
^{206}Pb	0.40	22.3142	22.0833
^{207}Pb	0.27	24.3830	24.1442
^{208}Pb	-0.28	1.4422	1.4255

Figure S3: MS[2] spectrum of Pb ions originated from Pb-EDTA complex breaking (NIST SRM 981, 500 µg/L). Deltas between expected and measured masses are ≤0.56 ppm. Ions having z=+1 are observed. Certified abundances are reported in [9] (Reed, W. P. National Institute of Standards & Technology Certificate of Analysis, Standard Reference Material 981; 1991). Flow: 15 µL/min. Injected volume: 250 µL. EDTA: 200 µM. Pb NIST 981: 500 µg/L. Solvent: H_2O:MeOH 1:1. Buffer: ammonium acetate 5 mM. Resolution: 15k. Microscan: 10. Quad. isol. window: 490-504 m/z. AGC target: 100%. Max inj. time: 100 ms. HCD: 200%. Scan window: 203.5-208.5 m/z.

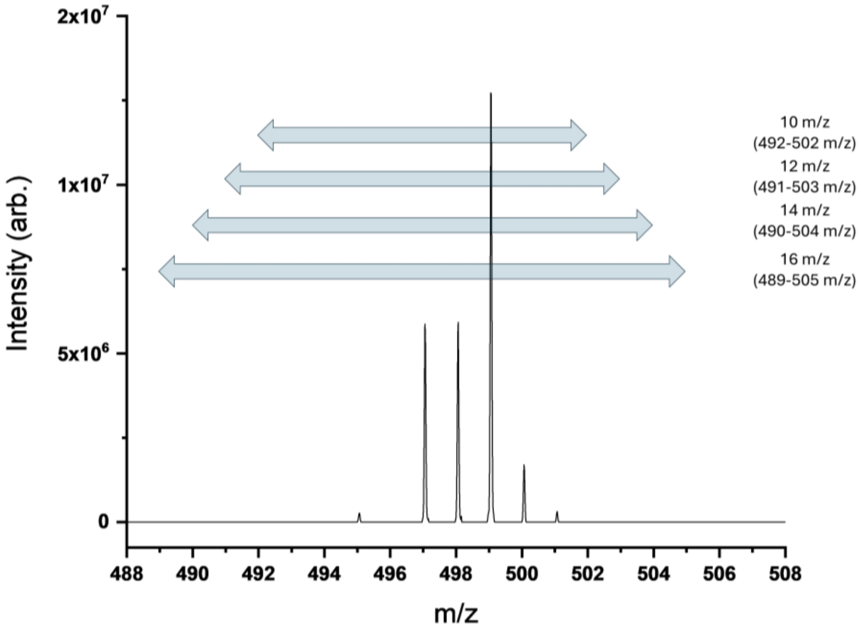

Figure S4. Width and position of tested quadrupole isolation windows. Isolation window must be large enough to include all precursor peaks.

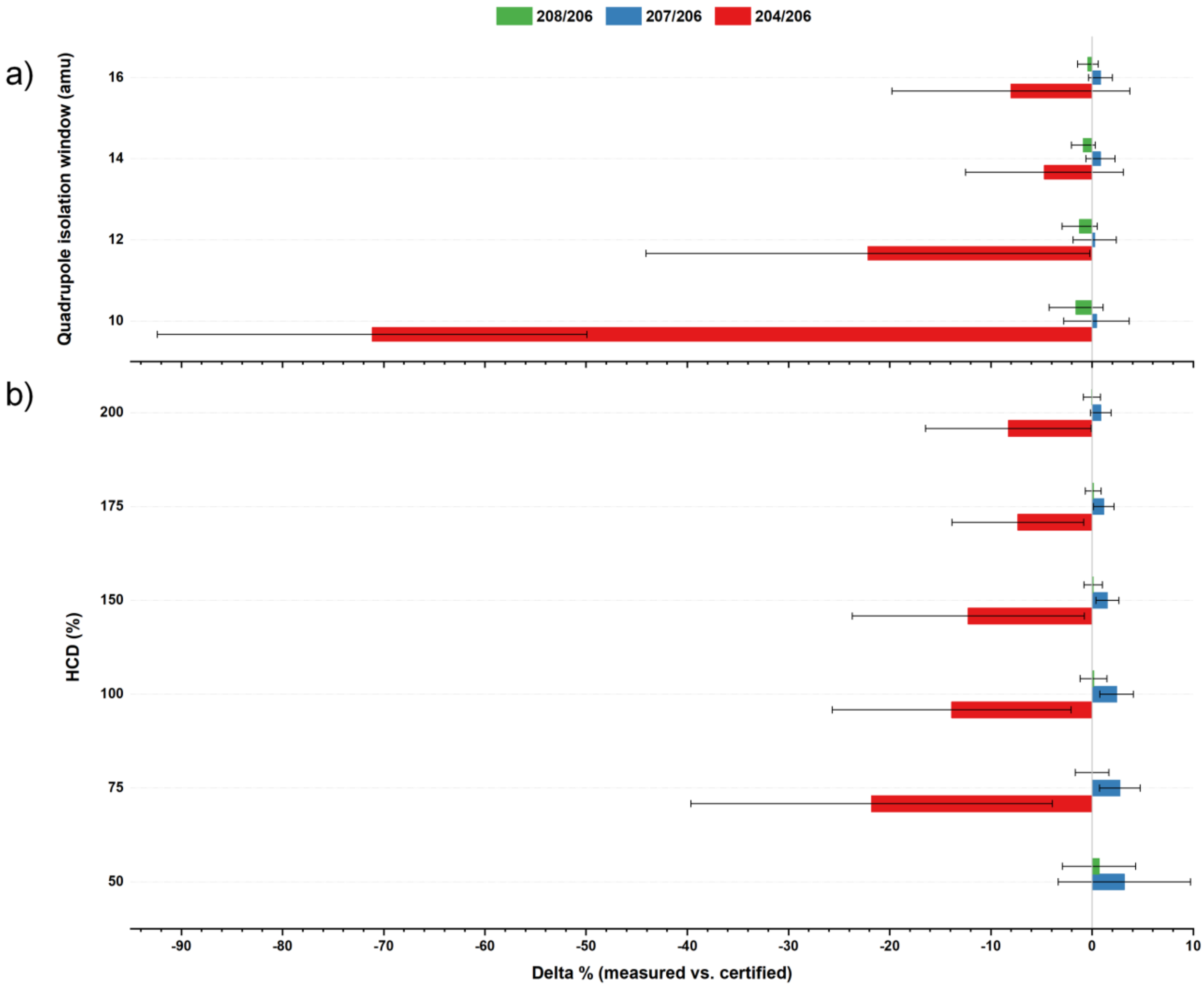

Figure S5. Effect of different parameters a) isolation window width, b) HCD collision energy on Pb ratios accuracy and precision using EDTA as the ligand. Common measurement conditions: Injected volume: 250 µL. Ligand: 50 µM. Pb NIST 981: 500 µg/L. Solvent: H_2O:MeOH 1:1. Resolution: 15k. Microscan: 10. AGC target: 100%. Max inj. time: 10 ms. Injection control: time. Scan window: 202-210 m/z. HCD 100% ≈ 68 V. Specific measurement conditions: a) HCD: 150%. b) Quad. isol. window: 493-504 m/z. Due to the low signal intensity, data are not available for isotope ^{204}Pb using a collisional energy of 50%.

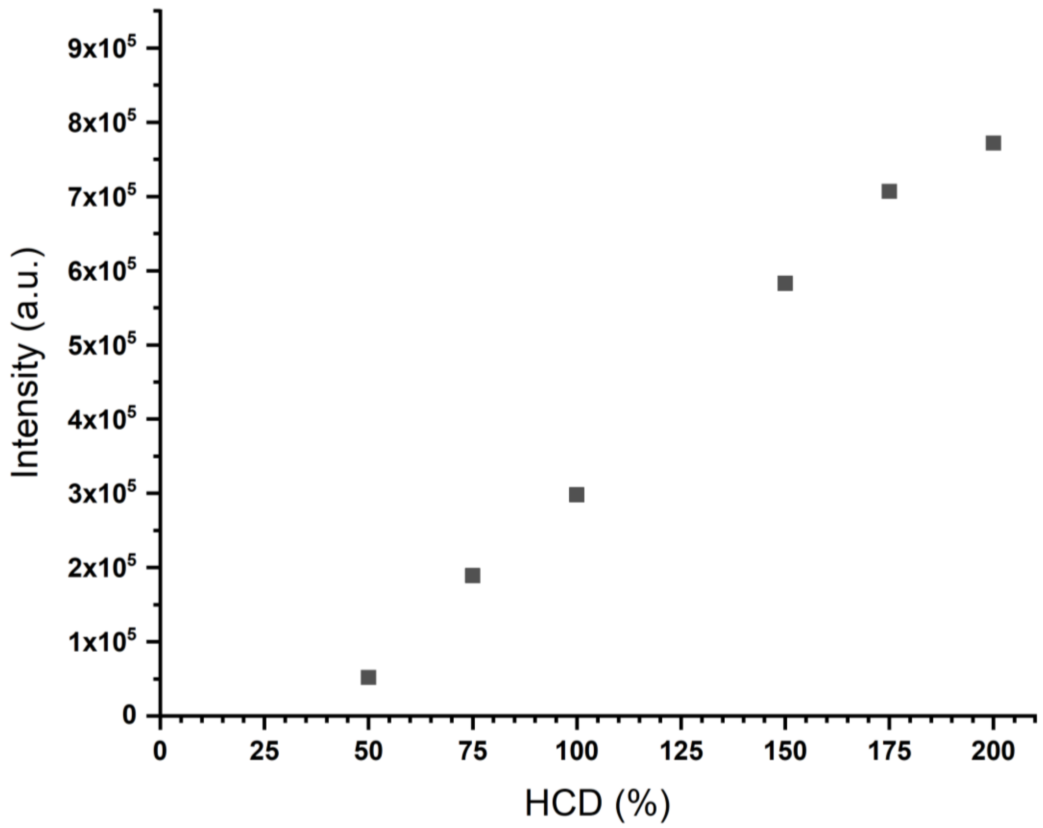

Figure S6. Effect of collision energy on ^{208}Pb signal intensity. HCD 100% ≈ 68 V (acceleration potential into the HCD cell).

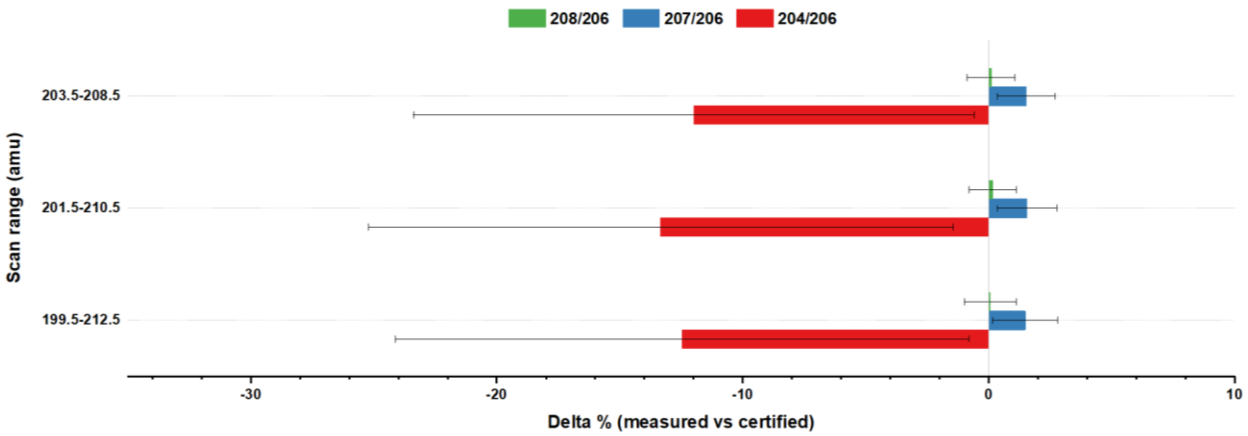

Figure S7. Effect of MS^2 scan range on ratio accuracy and precision using EDTA as ligand. Measurement conditions: Injected volume: 250 µL. Ligand: 50 µM. Pb NIST 981: 500 µg/L. Solvent: H_2O:MeOH 1:1. Resolution: 15k. Microscan: 10. Quad. isol. window: 493-504 m/z. AGC target: 100%. Max inj. time: 10 ms. Injection control: time. HCD: 150%.

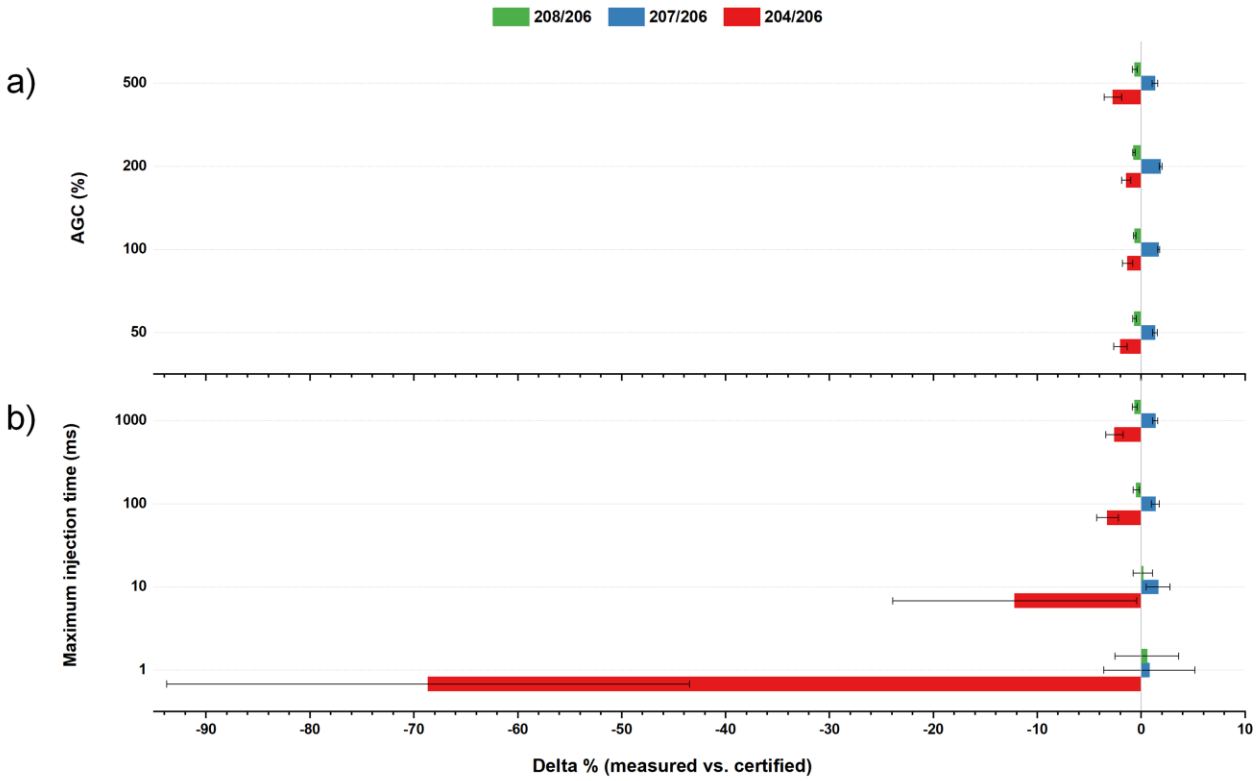

Figure S8. Effect of different parameters a) AGC™ target value, b) maximum injection time on Pb ratios accuracy and precision using EDTA as the ligand. Common measurement conditions: Injected volume: 250 µL. Ligand: 50 µM. Pb NIST 981: 500 µg/L. Solvent: H_2O:MeOH 1:1. Resolution: 15k. Microscan: 10. Quad. isol. window: 493-504 m/z. HCD: 150%. Scan window: 202-210 m/z. Specific measurement conditions: a) Max inj. time: 1000 ms. Injection control: AGC. b) AG C target: 1000%. Injection control: time.

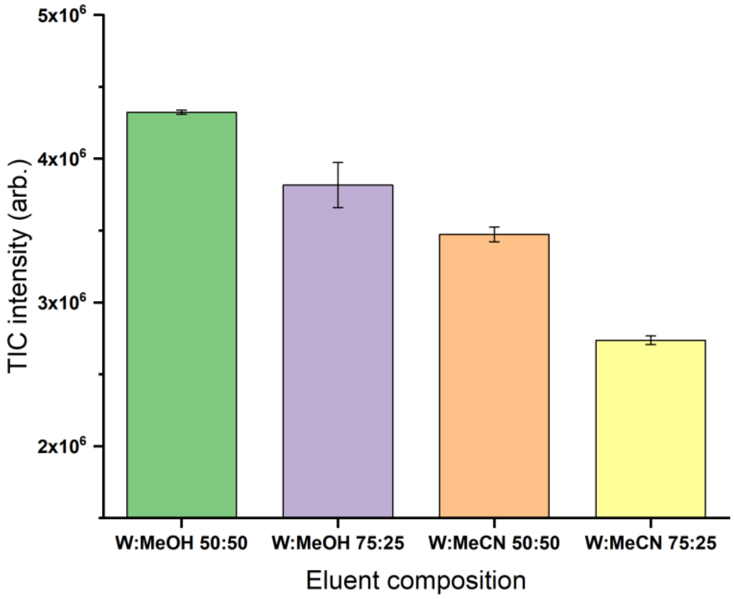

Figure S9. Effect of eluent composition on signal intensity. Average and SD of 3 replicates. TIC is the sum of the signals of all four stable isotopes of Pb.

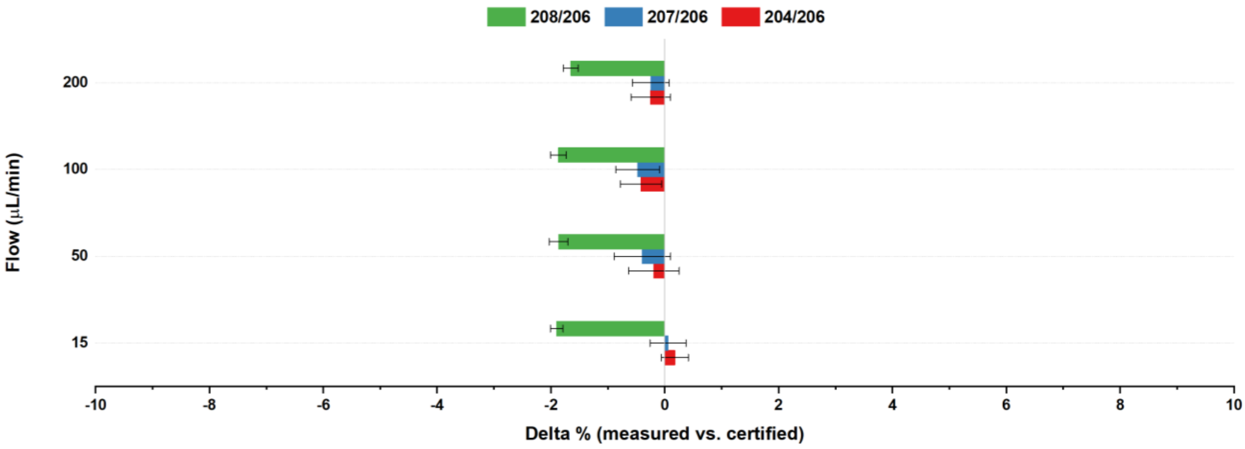

Figure S10. Effect of eluent flow on ratio accuracy and precision (EDTA). Injected volume: 250 µL (only for 15 µL/min flow condition) or 1000 µL. Ligand: 50 µM. Pb NIST 981: 500 µg/L. Solvent: H_2O:MeOH 1:1. Buffer: ammonium acetate 5 mM. Resolution: 15k. Microscan: 10. Quad. isol. window: 490-504 m/z. AGC target: 100%. Max inj. time: 100 ms. Injection control: time. HCD: 200%. Scan window: 203.5-208.5 m/z.

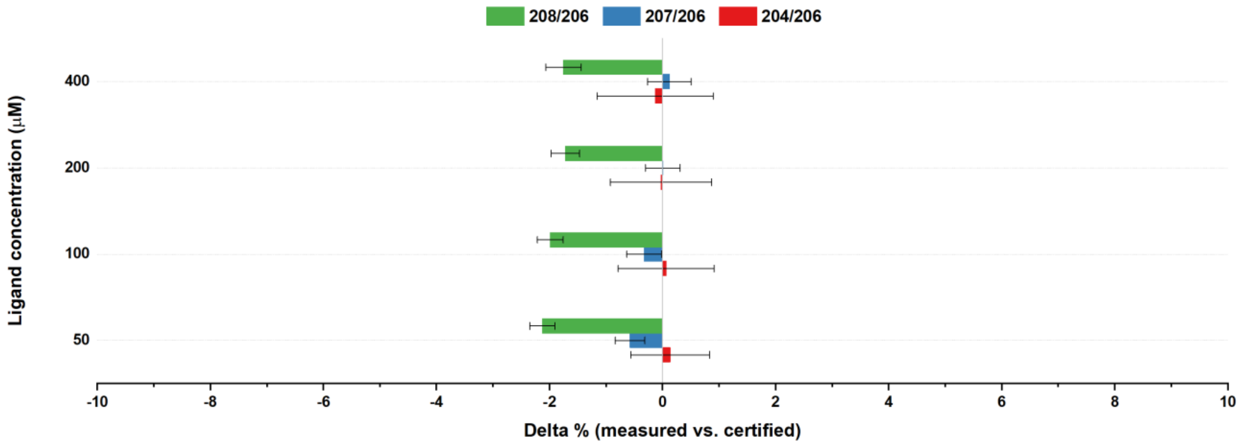

Figure S11. Effect of ligand concentration on ratio accuracy and precision (EDTA). Flow: 15 µL/min. Injected volume: 250 µL. Pb NIST 981: 500 µg/L. Solvent: H_2O:MeOH 1:1. Buffer: ammonium acetate 5 mM. Resolution: 15k. Microscan: 10. Quad. isol. window: 490-504 m/z. AGC target: 100%. Max inj. time: 100 ms. Injection control: time. HCD: 200%. Scan window: 203.5-208.5 m/z.

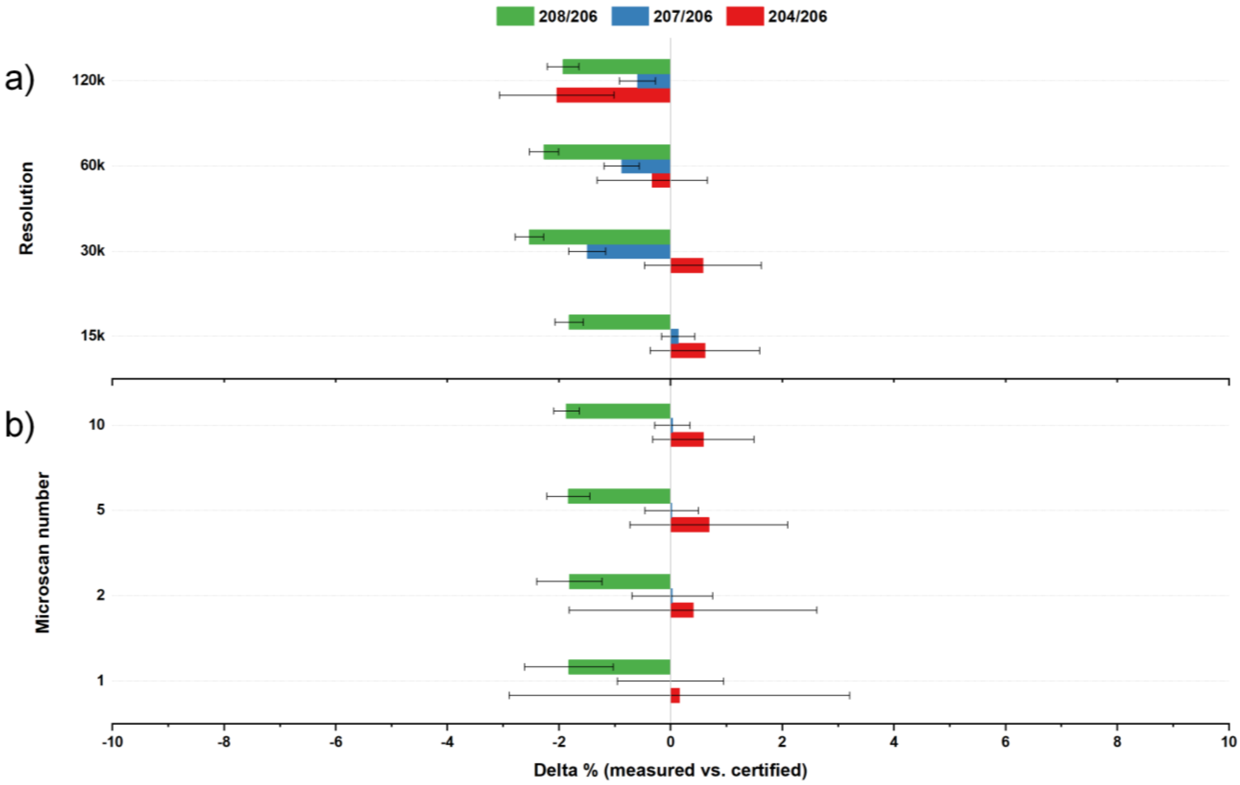

Figure S12. Effect of a) mass spectrometer resolution and b) microscan number on ratio accuracy and precision using EDTA as ligand. Common measurement conditions: Flow: 15 µL/min. Injected volume: 250 µL. Ligand: 200 µM. Pb NIST 981: 500 µg/L. Solvent: H_2O:MeOH 1:1. Buffer: ammonium acetate 5 mM. Quad. isol. window: 490-504 m/z. AGC target: 100%. Max inj. time: 100 ms. Injection control: time. HCD: 200%. Scan window: 203.5-208.5 m/z. Specific measurement conditions: a) Microscan number: 10; b) resolution: 15k.

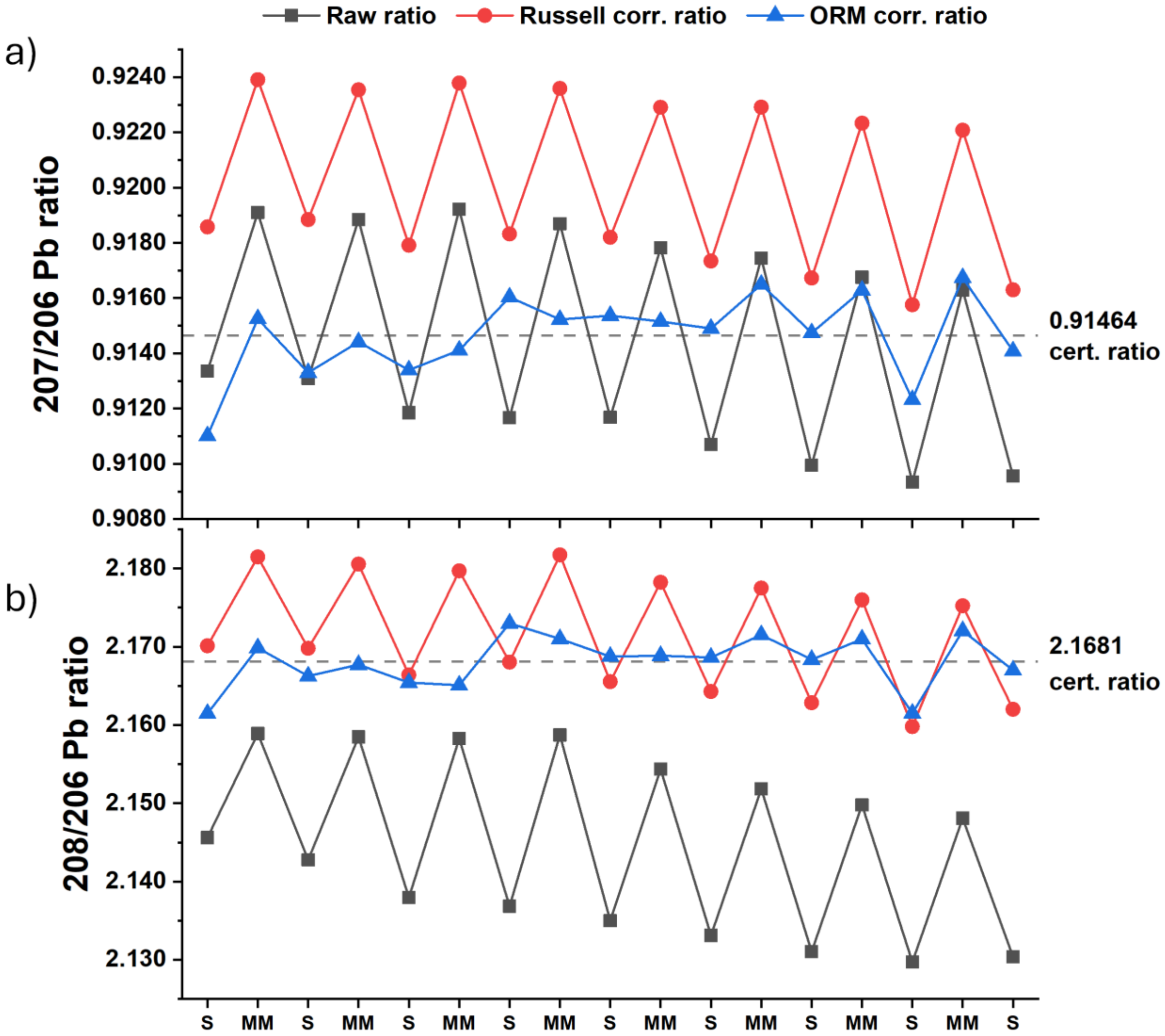

Figure S13. Measures were performed alternating conventional samples "S" (Pb NIST SRM 981 500 µg/L + Tl 500 µg/L) and matrix-modified samples "MM" (Pb NIST SRM 981 500 µg/L + Tl 500 µg/L + Ca 100 µM). Results were obtained applying different mass bias correction models using thallium as internal standard: a) data for $^{207}Pb/^{206}Pb$ ratio; b): data for $^{208}Pb/^{206}Pb$ ratio. Flow: 15 µL/min. Injected volume: 250 µL. EDTA: 200 µM. Solvent: H_2O:MeOH 1:1. Buffer: ammonium acetate 5 mM. Resolution: 15k. Microscan: 10. Quad. isol. window: 487-507 m/z. AGC target: 100%. Max inj. time: 100 ms. HCD: 200%. Scan window: 201.5-209.5 m/z.

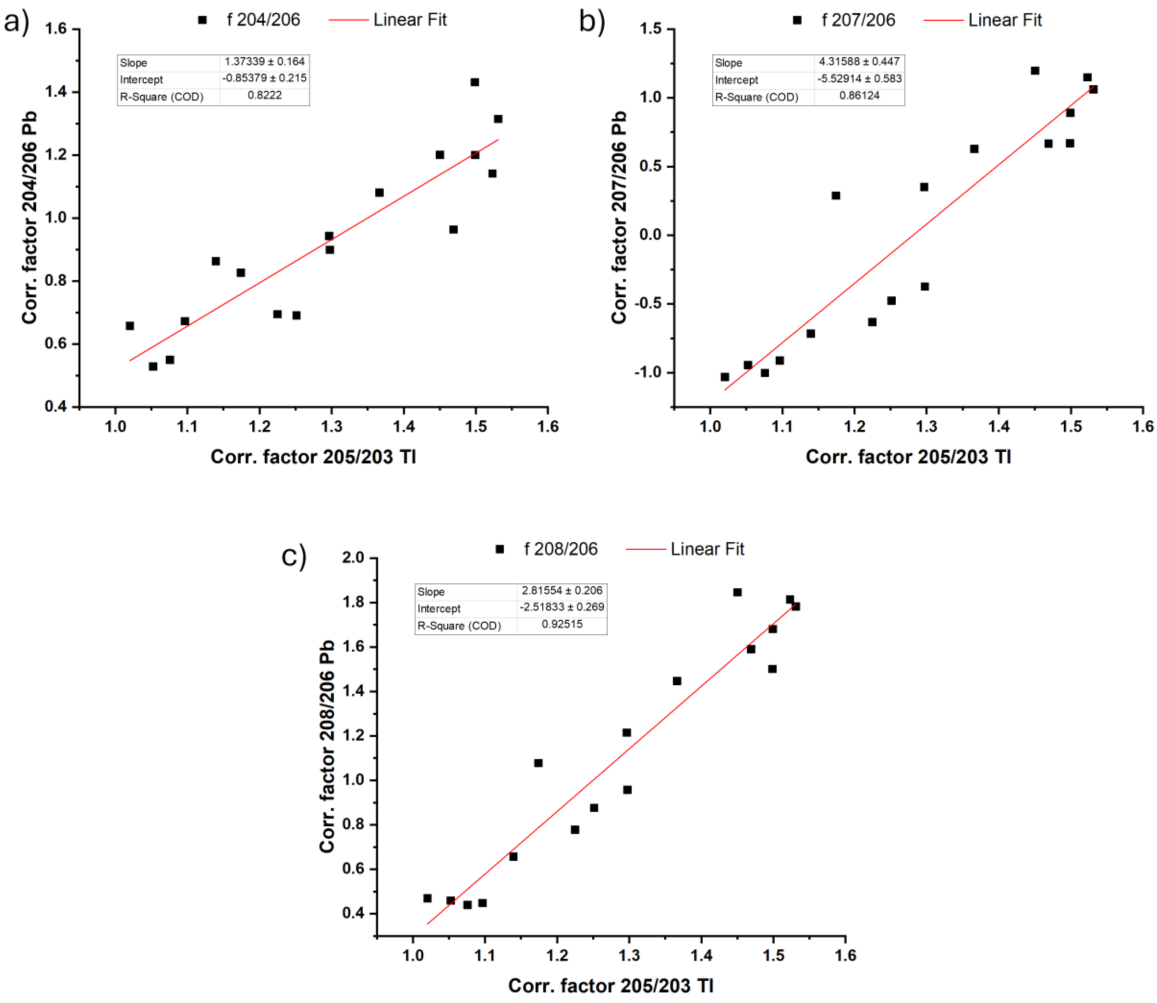

Figure S14: calibration curves for the ORM model. N= 17. As described in the Mass Bias correction section in the SI, data are corrected using a leave-one-out approach. A data is excluded from the calibration curve, and then the calibration curve is used to recalculate the correction factor for the excluded point. This scheme was reiterated for all the 17 data. a) correction factor for the 204/206 Pb IR. b) correction factor for the 207/208 Pb IR. c) correction factor for the 208/206Pb IR.

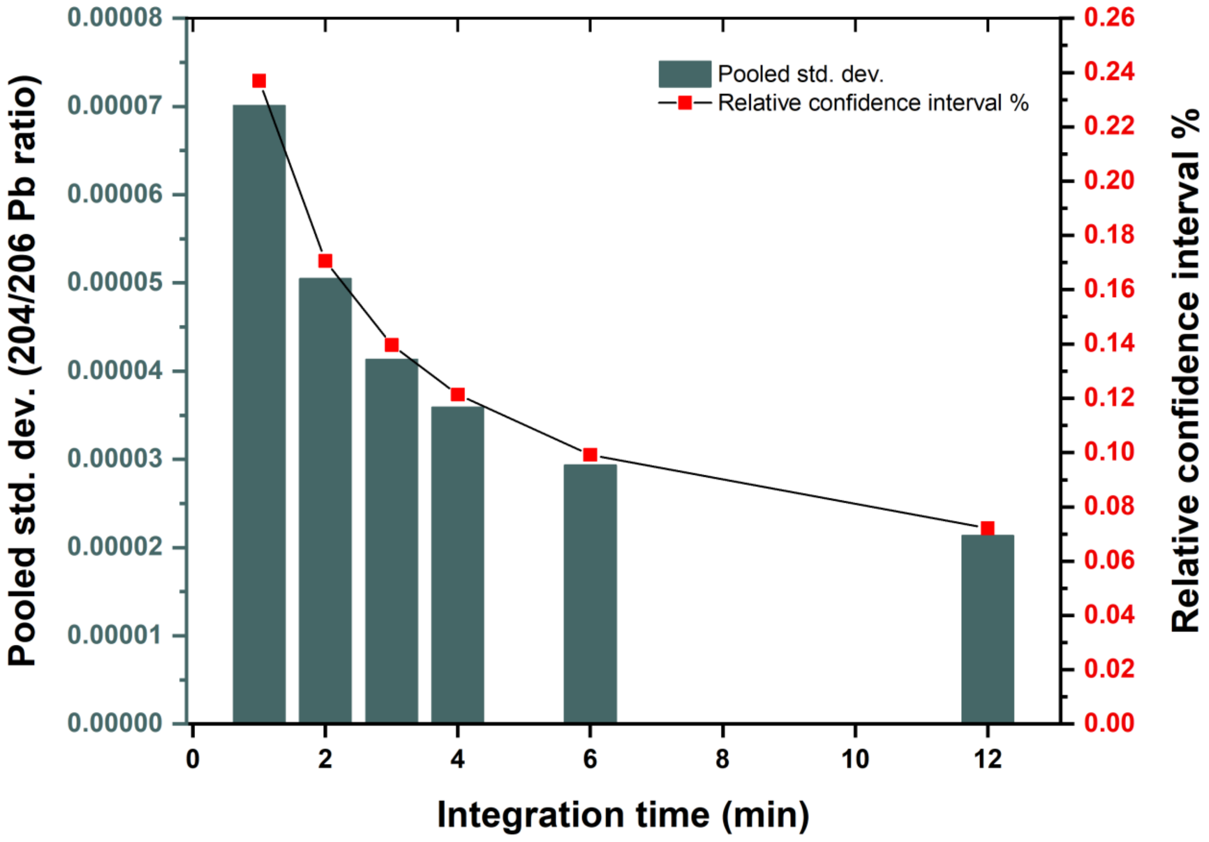

Figure S15. Effect of the integration time on the pooled standard deviation and relative percentual confidence interval (using a cov. factor = 2) for the isotope ratio $^{204}Pb/^{206}Pb$. Pooled SD are calculated keeping constant the total analysis time (12 minutes), but dividing this time in different integration times (e.g. for the integration time 1 min, we considered 12 intervals, for the integration time 2 min, we considered 6 intervals, etc.). Similar results were obtained for the isotope ratios $^{207}Pb/^{206}Pb$ and $^{208}Pb/^{206}Pb$.

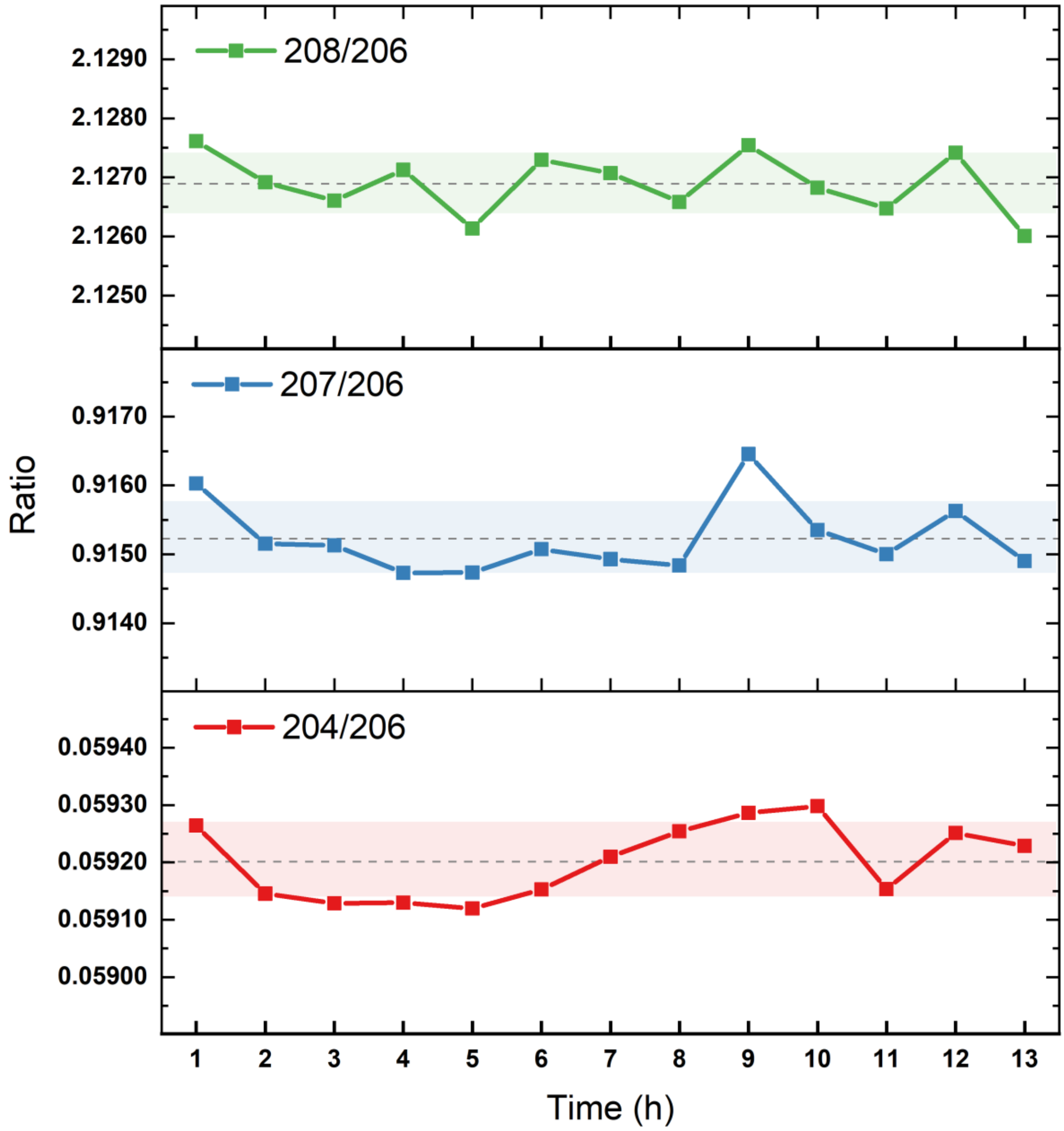

Figure S16. Temporal trend for different Pb ratios. 13 replicate measurements for NIST SRM 981 500 µg/L under optimized conditions. The dashed line represents the mean value, and the range of ±1 standard deviation from the mean is highlighted.

6. References of SI

(1) Monticelli, D.; Castelletti, A.; Civati, D.; Recchia, S.; Dossi, C. How to Efficiently Produce Ultrapure Acids. *Int. J. Anal. Chem.* **2019**, *2019*, 1–5. https://doi.org/10.1155/2019/5180610.

(2) Yang, L.; Tong, S.; Zhou, L.; Hu, Z.; Mester, Z.; Meija, J. A Critical Review on Isotopic Fractionation Correction Methods for Accurate Isotope Amount Ratio Measurements by MC-ICP-MS. *J. Anal. At. Spectrom.* **2018**, *33* (11), 1849–1861. https://doi.org/10.1039/c8ja00210j.

(3) Penanes, P. A.; Galán, A. R.; Huelga-Suarez, G.; Rodríguez-Castrillón, J. Á.; Moldovan, M.; Garcia Alonso, J. I. Isotopic Measurements Using ICP-MS: A Tutorial Review. *J. Anal. At. Spectrom.* **2022**, *37* (4), 701–726. https://doi.org/10.1039/d2ja00018k.

(4) Dunstan, L. P.; Gramlich, J. W.; Barnes, I. L.; Purdy, W. C. Absolute Isotopic Abundance and the Atomic Weight of a Reference Sample of Thallium. *J. Res. Natl. Bur. Stand.* **1980**, *85* (1), 1–10. https://doi.org/10.6028/jres.085.001.

(5) Eiler, J.; Cesar, J.; Chimiak, L.; Dallas, B.; Grice, K.; Griep-Raming, J.; Juchelka, D.; Kitchen, N.; Lloyd, M.; Makarov, A.; Robins, R.; Schwieters, J. Analysis of Molecular Isotopic Structures at High Precision and Accuracy by Orbitrap Mass Spectrometry. *Int. J. Mass Spectrom.* **2017**, *422*, 126–142. https://doi.org/10.1016/j.ijms.2017.10.002.

(6) Williams, T. J.; Hoegg, E. D.; Bills, J. R.; Marcus, R. K. Roles of Collisional Dissociation Modalities on Spectral Composition and Isotope Ratio Measurement Performance of the Liquid Sampling – Atmospheric Pressure Glow Discharge / Orbitrap Mass Spectrometer Coupling. *Int. J. Mass Spectrom.* **2021**, *464*, 116572. https://doi.org/10.1016/j.ijms.2021.116572.

(7) Beck, S. Fragmentation Behavior of EDTA Complexes under Different Activation Conditions. *J. Mass Spectrom.* **2021**, *56* (7), 1–16. https://doi.org/10.1002/jms.4775.

(8) Meier-Augenstein, W.; Schimmelmann, A. A Guide for Proper Utilisation of Stable Isotope Reference Materials. *Isotopes Environ. Health Stud.* **2019**, *55* (2), 113–128. https://doi.org/10.1080/10256016.2018.1538137.

(9) Reed, W. P. *National Institute of Standards & Technology Certificate of Analysis, Standard Reference Material 981*; 1991.
